# Immune Phenotype in Urothelial Carcinoma: From Tumor Biology to Therapeutic Stratification

**DOI:** 10.3390/cancers18152392

**Published:** 2026-07-24

**Authors:** Patricia Toquero, Lucía Castillo, Luis San José, Arantzazu Alfranca, Guillermo Celada, Clara Velasco, Laia Figols, Carlos Prada, María Pacheco, Pablo Gajate, Cristina Pernaut, Imanol Martínez, Ramón Colomer, Nuria Romero-Laorden

**Affiliations:** 1 Department of Medical Oncology, Hospital Universitario de La Princesa, 28006 Madrid, Spain; lcastillof@salud.madrid.org (L.C.); mariaisabel.pacheco.externo@salud.madrid.org (M.P.); rcolomer@seom.org (R.C.); 2 Cátedra UAM-Fundación Instituto Roche de Medicina Personalizada de Precisión, Universidad Autónoma de Madrid, 28049 Madrid, Spain; 3 Instituto de Investigación Sanitaria Princesa [IIS-Princesa], 28006 Madrid, Spain; lsanjose.hcsc@salud.madrid.org (L.S.J.); mariaaranzazu.alfranca@salud.madrid.org (A.A.); guillermo.celada@salud.madrid.org (G.C.); 4 Department of Urology, Hospital Universitario de La Princesa, 28006 Madrid, Spain; clara.velasco@salud.madrid.org; 5 Department of Immunology, Hospital Universitario de La Princesa, 28006 Madrid, Spain; laia.figols@salud.madrid.org; 6 Centro de Investigación Biomédica en Red de Enfermedades Cardiovasculares (CIBERCV), 28029 Madrid, Spain; 7 Department of Pathology, Hospital Universitario de La Princesa, 28006 Madrid, Spain; cprada@salud.madrid.org; 8 Department of Medical Oncology, Hospital Universitario Ramón y Cajal, 28034 Madrid, Spain; pablo.gajate@salud.madrid.org; 9 Department of Medical Oncology, Hospital Universitario Severo Ochoa, 28914 Madrid, Spain; cristina.pernaut@salud.madrid.org; 10 Department of Medical Oncology, Hospital Universitario Fundación Jiménez Díaz, 28040 Madrid, Spain; imanol.martinez@quironsalud.es

**Keywords:** urothelial carcinoma, tumor microenvironment, immune phenotype, tumor-infiltrating lymphocytes, immune checkpoint inhibitors, tertiary lymphoid structures, single-cell RNA sequencing, biomarkers, immunotherapy

## Abstract

Urothelial carcinoma (UC) is a biologically and clinically heterogeneous disease arising from the bladder and upper urinary tract. Immune checkpoint inhibitors (ICIs) have transformed treatment of advanced UC, yet durable responses remain limited to a minority of patients, underscoring the need to better understand the tumor immune microenvironment (TME). This narrative review examines the UC immune phenotype across disease stages and anatomical sites, integrating evidence from bulk and single-cell transcriptomics, spatial profiling, and translational studies. We describe the principal immune cell populations of the UC TME—including cytotoxic and regulatory T cells, macrophages, myeloid-derived suppressor cells, natural killer cells, B cells, and dendritic cells—and their relationship to established molecular subtypes. We review how this immune landscape evolves from non-muscle-invasive to metastatic disease, including the distinct contexture of upper tract UC and variant histology. We critically evaluate established ICI biomarkers (PD-L1, FGFR3, tumor mutational burden, mismatch repair deficiency) alongside emerging candidates—tumor-infiltrating lymphocyte density, HLA class I expression, tertiary lymphoid structures, and multiparameter transcriptomic scores—noting that most remain investigational and require prospective validation before clinical use. Finally, we address key biological, technical, and clinical barriers to this research and outline future directions in AI-assisted digital pathology and multimodal biomarker integration. A comprehensive characterization of UC immune phenotype is essential to guide rational, biomarker-driven patient selection and optimize next-generation immunotherapy strategies.

## Simple Summary

In this review, we comprehensively examine the immunological characteristics of urothelial tumors across all stages of the disease, anatomical locations, and tumor subtypes and critically evaluate both established and emerging biomarkers that could predict treatment response, such as immunotherapy. We also analyze the major biological and technical barriers hindering progress in this field and outline future directions, including the use of artificial intelligence and advanced tissue analysis platforms. By providing an integrated framework for the immunological landscape of urothelial carcinoma, this review aims to support the development of more precise, biology-based treatment strategies to improve patient outcomes.

## 1. Introduction

Urothelial carcinoma (UC) is the most common malignancy of the urinary tract, comprising tumors of the bladder, renal pelvis, ureter, and urethra. With an estimated 614,000 new cases and 220,000 deaths worldwide each year, UC represents a major public health burden [1]. Its clinical presentation spans a wide biological spectrum, from low-grade non-muscle-invasive bladder cancer (NMIBC), managed endoscopically with intravesical therapy, to muscle-invasive bladder cancer (MIBC), requiring radical cystectomy, and metastatic disease characterized by poor prognosis and limited treatment options until recently.

The introduction of immune checkpoint inhibitors (ICIs) targeting the PD-1/PD-L1 axis has transformed the management of locally advanced and metastatic UC (la/mUC) [2,3,4]. Five agents have received regulatory approval—atezolizumab, pembrolizumab, nivolumab, durvalumab, and avelumab—across multiple disease settings, including first-line (1L) cisplatin-ineligible patients, second-line platinum-refractory disease, adjuvant post-radical surgery, and maintenance therapy. More recently, the combination of enfortumab vedotin and pembrolizumab demonstrated superior overall survival over platinum-based chemotherapy in the first-line setting [2], reshaping the treatment paradigm. However, durable responses to ICI monotherapy occur in only 15–25% of patients [3], and robust predictive biomarkers capable of reliably selecting responding patients remain an unmet clinical need.

The fundamental determinant of ICI efficacy is the immune microenvironment: the composition, spatial organization, functional state, and dynamic evolution of immune cells within and around the tumor. UC offers a unique immunological setting—it is one of the most mutated solid tumors, historically responsive to BCG immunotherapy (the world’s first successful cancer immunotherapy), and characterized by pronounced molecular and immune heterogeneity driven by distinct transcriptomic subtypes [5,6]. Yet, despite this long-standing evidence for immune relevance, a comprehensive characterization of the UC immune phenotype across disease stages, anatomical sites, and histological subtypes is still lacking.

The emergence of high-resolution technologies—scRNA-seq, spatial transcriptomics, mass cytometry, and digital pathology—has provided unprecedented granularity in mapping the UC immune landscape. These tools have revealed the diversity of tumor-infiltrating lymphocyte (TIL) subsets, identified immunosuppressive myeloid populations, characterized tertiary lymphoid structures (TLSs), and linked immune phenotypes to molecular subtypes and clinical outcomes. Integrating this body of evidence with established and emerging biomarkers is essential to translate immune phenotyping into actionable clinical tools.

This review synthesizes the current knowledge on the immune phenotype in UC, structured around three main areas: (1) the biology and evolution of the immune microenvironment across disease stages and anatomical sites; (2) the prognostic and predictive value of established and emerging immune biomarkers; and (3) the principal barriers to immune phenotype research and future directions. By integrating evidence from seminal immunological studies, large transcriptomic datasets, and translational clinical trials, we aim to provide a framework for understanding and exploiting the immune landscape of UC to improve patient outcomes.

## 2. Materials and Methods

### 2.1. Study Design and Search Strategy

The present work was designed as a narrative review. A search was conducted in PubMed/MEDLINE combining MeSH terms (Group 1 terms; urothelial carcinoma: Carcinoma, Transitional Cell; Urinary Bladder Neoplasms; Non-Muscle Invasive Bladder Neoplasms, with Group 2 terms; tumor immunology: Immunity, molecular subtypes, Tumor-Infiltrating Lymphocytes, tumor microenvironment, MDSCs, TAMs, dendritic cells, Tregs, immune escape/tolerance, Immune Checkpoint Inhibitors, Immunotherapy). Case reports and book chapters were excluded; searches were restricted to Q1/Q2 journals, human studies published in English, and the last 10 years.

### 2.2. Study Selection

The initial search yielded 3248 records, reduced to 1928 after de-duplication (420 reviews). A first thematic screening excluded 282 articles (not urothelial-related, *n* = 16; case reports/letters, *n* = 70; no immunological focus, *n* = 196), leaving 1646 references. A second screening, focused on immunological relevance and currency (excluding the pre-2020 literature, purely clinical ICI studies, non-immunological biomarkers, and generalist reviews), narrowed this to 173. After a final relevance and content assessment, 79 articles were included.

**Figure 1:**
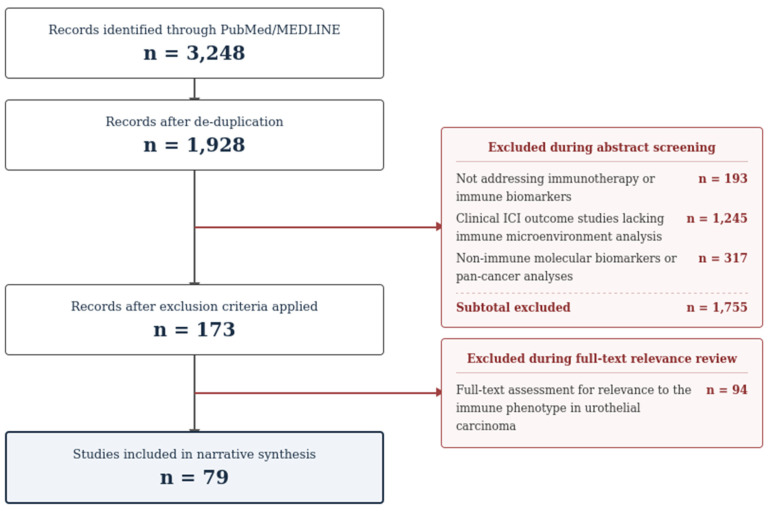
Flow diagram of the literature search and study selection process.

The initial search yielded 3.248 records, reduced to 1.928 after de-duplication. Then, study selection was performed by two independent reviewers. Due to the large number of articles identified, additional restricted exclusion criteria were applied: (1) studies not addressing immunotherapy or immune biomarkers (*n* = 193); (2) clinical ICI outcome studies lacking immune microenvironment analysis (*n* = 1.245); and (3) studies focused exclusively on non-immune molecular biomarkers or pan-cancer analyses without urothelial carcinoma-specific immune phenotype data (*n* = 317); using these criteria narrowed the search to 173 records. After a final relevance and content assessment, 79 articles were included (Figure 1). No quantitative synthesis or meta-analysis was performed. 

## 3. The Immune System in the Urothelial Carcinoma Setting

### 3.1. Evidence for Immune Involvement: Historical Context

The intimate relationship between the immune system and UC was first formally recognized with the introduction of intravesical Bacillus Calmette–Guérin (BCG) by Morales and colleagues in 1976. BCG immunotherapy is considered the standard-of-care adjuvant treatment for high-risk NMIBC. Its therapeutic effect is mediated through the induction of a local innate and adaptive immune response characterized by TH1 cytokine secretion, the recruitment of T cells and NK cells, and direct antitumor cytotoxicity [7]. The success of BCG established proof-of-concept that UC is an immunologically responsive disease and set the stage for the modern era of checkpoint immunotherapy.

Early histopathological observations documented the prognostic significance of immune infiltrates in UC. Increased CD8+ T-cell density within the tumor and its stroma was associated with improved survival following radical cystectomy [8]. Elevated stromal lymphocytic infiltrates were paradoxically linked to worse outcomes in certain contexts, potentially reflecting Treg-mediated immunosuppression or immune exclusion [8,9]. More recently, the Immunoscore—a quantitative measure of CD3+ and CD8+ T-cell densities at the invasive margin and tumor center—has been validated as an independent prognostic factor in MIBC patients undergoing radical cystectomy, adding value beyond pathological stage [10].

The advent of large-scale transcriptomic profiling, culminating in the TCGA bladder cancer dataset and subsequent molecular classification systems, revealed that immune infiltration is not random but tightly linked to molecular subtype, with profound implications for ICI responsiveness [5].

### 3.2. Immune Cell Populations in the UC Microenvironment

The UC TME comprises a complex ecosystem of immune cells whose relative abundance, spatial distribution, and functional state vary across tumor subtypes, disease stages, and anatomical locations.

#### 3.2.1. Cytotoxic CD8+ T Cells

CD8+ cytotoxic T lymphocytes (CTLs) represent the primary effectors of anti-tumor immunity and are the key target of PD-1/PD-L1 checkpoint blockade. In UC, higher intratumoral CD8+ T-cell density is consistently associated with improved prognosis and ICI response [8,11]. Single-cell studies have refined this picture, identifying distinct CD8+ subpopulations with divergent functional states: effector/memory T cells (Teff/Tem), tissue-resident memory T cells (Trm; CD103+CD69+), precursor-exhausted T cells (Tpex; TCF-1+PD-1+), and terminally exhausted T cells (Tex; TOX+TIM-3+LAG-3+) [12,13]. Tissue-resident memory T cells—epigenetically cytotoxic and bearing signs of early exhaustion—are particularly enriched in bladder UC and have been proposed as key responders to PD-1 blockade [13]. Conversely, terminally exhausted CD8+ T cells, characterized by co-expression of TOX, TIM-3, LAG-3, and loss of TCF-1, are resistant to checkpoint reinvigoration and correlate with poor ICI response [14].

CD8+ T cells expressing ILT2 (LILRB1), a receptor inhibited by HLA-G, represent a distinct intratumoral cytotoxic population identified in bladder UC. HLA-G overexpression on tumor cells engages ILT2+CD8+ T cells, selectively suppressing their anti-tumor activity and constituting a non-canonical immune escape mechanism [15]. This finding has direct therapeutic implications, as HLA-G/ILT2-targeted approaches are under early clinical investigation.

#### 3.2.2. CD4+ T Cells and Regulatory T Cells

CD4+ helper T cells play a multifaceted role in UC immunity. Tumor-reactive CD4+ T cells, including KLRG1+ cytotoxic CD4+ cells, contribute directly to anti-tumor responses and provide critical help for CD8+ CTL maintenance [16]. Follicular helper T cells (Tfh) within tumor-draining lymph nodes and tertiary lymphoid structures (TLSs) promote B-cell maturation and the production of anti-tumor antibodies [17].

Regulatory T cells (Tregs; CD4+FoxP3+CD25+) are consistently enriched in the UC TME and are associated with immune suppression and poor prognosis [18]. The CD8/Foxp3 ratio, a composite immunohistochemical metric applicable to routine FFPE tissue, has validated prognostic and predictive value in NMIBC, where a reduced ratio is associated with higher recurrence risk and poorer response to BCG immunotherapy [19]; its predictive value for ICI response in MIBC has not yet been formally established and merits dedicated validation. Notably, a subset of PD-L1-expressing Tregs is enriched following BCG therapy in NMIBC, potentially contributing to BCG resistance through the suppression of anti-tumor CTL responses [19].

#### 3.2.3. Tumor-Associated Macrophages

Tumor-associated macrophages (TAMs) represent the most abundant innate immune population in the UC TME and are predominantly polarized towards an immunosuppressive M2-like phenotype [20]. High TAM infiltration, particularly CD163+ M2 macrophages, is associated with advanced stage, lymphovascular invasion, and poor prognosis in MIBC [20,21]. TAMs contribute to immune evasion through multiple mechanisms: PD-L1 upregulation on their surface, production of immunosuppressive cytokines (IL-10, TGF-β), promotion of angiogenesis, and physical exclusion of cytotoxic T cells from the tumor nest [22].

Single-cell and spatial analyses have revealed remarkable macrophage heterogeneity in UC, identifying distinct subpopulations including galectin-9-expressing immunoevasive TAMs, DC-SIGN+ macrophages capable of stimulating T-cell responses when reprogrammed, and SPP1+ immunosuppressive macrophages enriched in treatment-resistant tumors [6,13]. The TREM2+ macrophage subset, associated with lipid metabolism and immune exclusion, has been identified in UC as a potential therapeutic target [23]. However, the binary M1/M2 classification framework, while conceptually useful, inadequately captures the true heterogeneity and functional plasticity of TAMs, and clinical trials of TAM-targeting agents (CSF1R inhibitors, CD47 blockade) have repeatedly failed to demonstrate durable clinical benefit—in part because the depletion of one macrophage subset is often compensated by the recruitment of alternative immunosuppressive myeloid populations [24]. This underscores that therapeutic targeting of TAMs in UC will likely require precision strategies informed by single-cell-resolved TAM heterogeneity rather than broad M1/M2-directed approaches [24].

#### 3.2.4. Myeloid-Derived Suppressor Cells

MDSCs comprise a heterogeneous population of immature myeloid cells with potent immunosuppressive activity, subdivided into polymorphonuclear (PMN-MDSCs; CD15+CD33+HLA-DR−) and monocytic (M-MDSCs; CD14+HLA-DR−) subsets. In UC, elevated circulating and intratumoral MDSC levels are associated with advanced disease, platinum resistance, and poor ICI response [6,25]. MDSCs suppress T-cell function through arginase-1-mediated arginine depletion, reactive oxygen species production, and PD-L1/PD-L2 expression [6]. Accumulation of MDSCs induced by low-level IL-6 signaling—a mechanism operative in UC—has been linked to adverse prognosis and constitutes a potential biomarker and therapeutic target [26].

The combination of MDSC targeting with ICI has shown preclinical synergy in FGFR3-mutant bladder cancer models, where FGFR3 inhibition reduces MDSC recruitment and augments anti-PD-1 efficacy [27]. Neoadjuvant chemotherapy has been shown to modulate MDSC populations in MIBC, potentially improving the immune contexture ahead of ICI or surgery [28].

#### 3.2.5. Natural Killer Cells

NK cells are innate lymphocytes capable of MHC class I-unrestricted tumor killing, particularly relevant in tumors with HLA-I downregulation. In UC, NK cell infiltration and the DNAM-1/KIR inhibitory receptor ratio—a measure of NK cell education and activation state—have been linked to prognosis and anti-tumor immunity [29]. TGF-β, abundantly produced in the UC TME, impairs NK cell cytotoxicity by downregulating activating receptors such as NKG2D and DNAM-1 [30]. Interestingly, sex-dependent differences in NK cell infiltration have been observed in UC, with female patients showing distinct NK cell-mediated immune responses that may partially explain sex-based differences in ICI outcomes [31].

#### 3.2.6. B Cells and Tertiary Lymphoid Structures

B cells and plasma cells have emerged as important components of the UC immune response. Intratumoral B cells, particularly those organized into TLSs—ectopic lymphoid aggregates containing germinal center-like structures—are associated with improved prognosis and ICI response in MIBC [32,33]. TLSs provide a local site of T-cell priming, B-cell maturation, and antibody production, effectively functioning as immune-activating niches within the tumor. CXCL13, produced by follicular helper T cells and TLS-associated B cells, has been proposed as a practical serum and tissue surrogate for TLS presence and may be related to the responsiveness to ICI [34].

Conversely, immunosuppressive regulatory B cells (Bregs) producing IL-10 are enriched in advanced UC and suppress anti-tumor T-cell responses, potentially contributing to ICI resistance [35]. The emerging role of atypical B cells in BCG failure in NMIBC further highlights the complex and context-dependent functions of B cells in UC immunity [36].

#### 3.2.7. Dendritic Cells

Dendritic cells (DCs) are essential professional antigen-presenting cells that bridge innate and adaptive immunity. In UC, conventional DCs (cDC1s; CD103+XCR1+) are critical for the cross-presentation of tumor antigens to CD8+ T cells and are enriched in tumors with hot immune phenotypes [37]. DC-SIGN+ (CD209+) TAMs with DC-like properties have been identified in MIBC; their functional reprogramming towards an antigen-presenting state is associated with improved outcomes and may be therapeutically exploitable [6,13]. The LAMP3+ mature migratory DC subset, enriched within TLS, plays a key role in T-cell priming and ICI response in multiple solid tumors including UC [37].

### 3.3. Relationship with Molecular Subtypes

The UC immune phenotype is not randomly distributed but is tightly coupled to the underlying molecular architecture of the tumor. Multiple complementary classification systems—TCGA (2014, 2017), Lund (2017), and the MIBC Consensus (2020)—converge on a fundamental distinction between luminal and basal/squamous subtypes, each with characteristic immune features [5,38,39] (Table 1).

Luminal papillary tumors, enriched for FGFR3 alterations and characterized by urothelial differentiation, consistently exhibit a cold or immune-desert phenotype with low TIL density and minimal immune infiltration. FGFR3 signaling actively suppresses the anti-tumor immune response through multiple mechanisms: the downregulation of antigen presentation, induction of MDSC recruitment, degradation of PD-L1 via NEDD4, and promotion of an immunosuppressive cytokine milieu [40,41]. Consequently, luminal papillary tumors have been associated with poorly respond to ICI monotherapy than other subtypes, although this association is probabilistic rather than absolute and is modulated by co-existing factors such as TMB, PD-L1 expression, and metastatic site (Section 4.2.2) [40].

Basal/squamous tumors, characterized by expression of basal epithelial markers (KRT5/6, KRT14, CD44) and high genomic instability, consistently display a hot or inflamed immune phenotype with abundant CD8+ TIL infiltration, elevated PD-L1 expression, and active immune response. Despite this immune activation, the co-enrichment of immunosuppressive Tregs and TAMs creates a functional checkpoint that limits spontaneous immune control but makes these tumors more likely to respond to ICI therapy [5,42], although further prospective studies are needed.

Neuronal tumors represent the most immunologically inert subtype, resembling a cold/immune-desert phenotype virtually devoid of TILs and characterized by strong neuroendocrine programme expression. These tumors are associated with the worst prognosis and show minimal ICI benefit [43].

Stroma-rich tumors are characterized by abundant cancer-associated fibroblast (CAF) infiltration creating a physical barrier to T-cell infiltration—the paradigmatic immune-excluded phenotype—with TGF-β-mediated suppression of effector immunity [44].

It should be noted that this subtype classification, while clinically useful, is not strictly static: molecular subtypes show significant overlap at the boundary, and luminal tumors can acquire basal-like features under therapeutic pressure—including chemotherapy and ICI exposure—reflecting underlying tumor plasticity rather than a fixed cell-of-origin state. This interconversion has direct implications for biomarker interpretation, as a single pre-treatment classification may not remain representative of the tumor’s immune phenotype at the time of treatment failure or progression.

### 3.4. Dynamic Changes During Cancer Progression: NMIBC → MIBC → Metastasis

The immune landscape of UC is not static but undergoes profound remodeling as the disease progresses from the superficial non-invasive stage to muscle invasion and ultimately metastasis—a process driven by tumor-intrinsic immunoediting and extrinsic pressure from the microenvironment. 

It should be noted that the majority of studies described below are based on single-timepoint or paired pre-/post-treatment biopsies rather than longitudinal sampling of the same tumor clones; as discussed in Section 5.1, this design limitation makes it difficult to fully distinguish genuine treatment-induced immune remodeling from spatial sampling variability or selection of pre-existing tumor subclones.

#### 3.4.1. Non-Muscle-Invasive Bladder Cancer

In NMIBC, the immune microenvironment is characterized by a relatively preserved capacity for immune recognition, maintained in part by a largely intact urothelial barrier and recurrent antigen exposure. High-grade T1 and carcinoma in situ (CIS) lesions, however, already display evidence of immune editing: PD-L1 upregulation, Treg infiltration, and early signs of T-cell dysfunction [19]. The immune phenotype of NMIBC is predictive of BCG response: tumors with inflamed, CD8+-enriched TME are more likely to respond, while those with immune-desert or excluded phenotypes—enriched for FGFR3 mutations and luminal markers—tend to be BCG-unresponsive [20].

Single-cell and spatial analyses of NMIBC have revealed dynamic shifts in immune cell composition during BCG therapy, with the early expansion of NK cells and CTLs followed by Treg-mediated suppression in non-responding patients [45]. Temporal dynamics of immune cell infiltration during BCG therapy constitute a clinically relevant window for biomarker capture and combination therapeutic targeting.

#### 3.4.2. Muscle-Invasive Bladder Cancer

The transition from NMIBC to MIBC is associated with significant immune remodeling. Loss of HLA class I expression—a mechanism of T-cell immune escape—occurs progressively with stage and is more prevalent in MIBC than NMIBC [45]. Neoadjuvant platinum-based chemotherapy, the standard perioperative treatment for MIBC, has been shown to modulate the immune microenvironment, though reported effects are heterogeneous and often transient across studies: while some cohorts show increased CD8+ TIL density and reduced MDSC burden, others report MDSC expansion and PD-L1 upregulation on tumor cells consistent with an adaptive immunosuppressive response rather than a uniformly favorable ‘priming’ effect [28,46]. This heterogeneity underscores that chemotherapy-induced TME remodeling should not be assumed to consistently favor ICI combination strategies. Indeed, pathological complete response (pCR) following neoadjuvant chemoimmunotherapy correlates with a highly inflamed TME at baseline and on-treatment, validating immune phenotype as a surrogate of therapeutic efficacy [47].

In the perioperative MIBC setting, immune gene signatures—rather than molecular subtype alone—are the strongest predictors of benefit from neoadjuvant ICI-based therapy, as demonstrated in the ABACUS and PURE-01 neoadjuvant cohorts [47], although molecular classification more broadly continues to inform neoadjuvant treatment selection across chemotherapy- and immunotherapy-based approaches [48]. The T-cell-to-stroma enrichment (TSE) score, a gene expression-based metric quantifying the balance between T-cell infiltration and stromal exclusion, appears to be a strong predictor of ICI response independently of molecular subtype across MIBC cohorts [33].

#### 3.4.3. Metastatic Disease

In metastatic UC, the immune phenotype is further complicated by site-specific differences in immune infiltration, systemic immunosuppression, and prior treatment history. Liver metastases are characterized by a particularly immunosuppressive microenvironment—poor TIL infiltration, high TAM density, and impaired ICI response—which correlates with reduced overall survival in ICI-treated patients [49]. Bone and lymph node metastases display intermediate immune phenotypes, while lung metastases may retain higher immune activity [49].

Spatial immunophenotyping studies of matched primary tumors and distant metastases in UC have demonstrated that the immune phenotype of metastatic sites—rather than the primary tumor—may provide a stronger predictor of ICI response [50]. This observation has profound implications for biopsy strategy in clinical trials and argues for the sampling of metastatic lesions when feasible. Clonal diversity within the tumor, including immune cell clonal heterogeneity, further shapes ICI response in the metastatic setting [20].

Beyond tumor-intrinsic factors, the dynamic remodeling of the immune microenvironment induced by systemic therapies represents an increasingly recognized determinant of response in metastatic UC. Antibody–drug conjugates, most notably enfortumab vedotin, have been shown to promote immunogenic cell death, facilitate dendritic cell maturation, and enhance cytotoxic CD8+ T-cell infiltration—effects that provide a compelling mechanistic basis for the observed synergy with immune checkpoint inhibition [2] and highlight the critical role of treatment-induced immunomodulation in the context of combination strategies.

### 3.5. Differences Between Bladder Cancer and Upper Tract Urothelial Carcinoma

Upper tract urothelial carcinoma (UTUC) accounts for approximately 5–10% of UC cases and has historically been understudied relative to bladder UC. UTUC is not a single biological entity: sporadic, Lynch syndrome-associated, renal pelvis, and ureteral tumors differ in mutational landscape and immune contexture, and the evidence base for UTUC-specific immune biomarkers remains limited, heterogeneous, and predominantly retrospective; direct extrapolation from bladder UC data should therefore be avoided. Emerging transcriptomic and spatial analyses reveal a distinct immune contexture in UTUC compared to bladder cancer, with significant clinical implications.

UTUC is enriched for the luminal papillary molecular subtype, characterized by a T-cell depleted, immune-cold TME with high prevalence of FGFR3 alterations [16]. These associations derive largely from small, retrospective, single-institution cohorts and should be interpreted as hypothesis-generating; whether they hold consistently across sporadic versus Lynch-associated, or renal pelvis versus ureteral disease, remains to be established. This immunologically quiescent phenotype in the majority of UTUC likely contributes to the observed lower response rates to ICI compared to bladder UC in retrospective series [51]. However, a minority of UTUC cases—particularly those with MMR deficiency, high TMB, or basal molecular features—display inflamed immune phenotypes with potential for ICI benefit.

Intratumoral IDO-1 expression, a key tryptophan-catabolizing enzyme mediating T-cell suppression, has been proposed as an immune classifier in UTUC, identifying a subset with high immunosuppressive activity despite some degree of T-cell infiltration [52]. Comprehensive integrative profiling of UTUC has identified immune-hot, immune-warm, and immune-cold clusters with distinct survival outcomes, supporting the applicability of immune phenotype classification to UTUC [53].

PD-L1 expression in UTUC, assessed by urine cytology and tissue biopsy, shows moderate predictive value for ICI response, with higher concordance between primary and metastatic sites compared to bladder UC [54]. The unique anatomical and histological characteristics of UTUC—including the thinner urothelial mucosa, proximity to renal parenchyma, and specific mutational landscape (Lynch syndrome association, CDKN2A deletion)—may further modulate the immune response.

### 3.6. Rare Histological Subtypes and Neuroendocrine Urothelial Carcinoma

Variant histologies of UC—including sarcomatoid, plasmacytoid, micropapillary, squamous, and glandular differentiation—are associated with aggressive clinical behavior, but biological immune phenotype, PD-L1 expression, immune infiltration, clinical response to ICI, and intrinsic prognosis do not necessarily align and should be considered as distinct, separately evidenced dimensions rather than a single composite profile; evidence for each is largely retrospective and derived from small cohorts [43,55].

Sarcomatoid UC is characterized by loss of epithelial identity, acquisition of mesenchymal features, and an immune-excluded or immune-desert phenotype with poor TIL infiltration but paradoxically high PD-L1 stromal expression. Retrospective data suggest these tumors may respond to ICI despite their immunologically cold appearance, possibly through non-T-cell-mediated immune mechanisms [55]. Squamous cell differentiation, common in bladder UC (10–20% of cases), is associated with a basal/squamous molecular subtype and an inflamed immune phenotype, potentially conferring ICI sensitivity; however, HER2 amplification co-occurring in this subtype may modulate immune responses [56].

Small cell/neuroendocrine UC represents the most aggressive and immunologically inert variant, characterized by rapid proliferation, early distant metastasis, and virtual absence of TIL infiltration. The “immune-desert” phenotype of neuroendocrine UC, driven by epigenetic silencing of antigen presentation machinery and strong expression of neuroendocrine transcriptional programmes, renders these tumors largely refractory to ICI as monotherapy. Combination strategies with DNA-damaging agents, epigenetic modulators, or DLL3-targeted therapies are under investigation [43].

### 3.7. Sex- and Age-Related Differences in Immune Phenotype

Biological sex modulates immune responses through differential expression of sex hormone receptors on immune cells and sex-linked differences in immune gene regulation. In UC, males have approximately fourfold higher incidence, but females present with more advanced stage at diagnosis, higher genomic complexity—both important confounders that complicate causal interpretation of sex-based differences in ICI outcomes.

A systematic review and network meta-analysis pooling urothelial and renal cell carcinoma found that the direction of the sex-survival association with ICI/ADC therapy varied by treatment setting—a female advantage in the adjuvant setting and a male advantage in second-line therapy—rather than a uniform female survival advantage, and did not adjust for stage, age, or hormonal status at the individual-patient level [57].

Single-cell analyses have identified sex-specific differences in TIL composition, T-cell clonality, and B-cell gene signatures within bladder tumors. In a single retrospective, hypothesis-generating study of 348 ICI-treated patients and a non-ICI-treated comparator cohort, sex-based stratification of survival following ICI is associated with intratumoral expression of a B-cell gene signature [31]; however, this analysis did not control for stage, age, hormonal status, or prior treatment history, and its findings should be regarded as exploratory rather than establishing a causal mechanism. Estrogen receptor signaling has been proposed, largely from pan-cancer data outside UC, to upregulate antigen presentation and immune surveillance—a plausible but unvalidated hypothesis in UC specifically. Real-world clinical evidence on sex-based ICI outcomes remains conflicting, with some analyses reporting a female survival advantage [57] and others reporting inferior outcomes in women treated with pembrolizumab [58], underscoring that this remains an open question requiring confounder-adjusted, prospective study.

Age-related immunosenescence—characterized by reduced naive T-cell output, increased Treg activity, and impaired DC function—may affect ICI responsiveness in elderly UC patients, who represent a significant proportion of the overall UC population. However, clinical evidence does not systematically support reduced ICI efficacy in older patients, and baseline performance status appears to be a more robust predictor than chronological age per se [59].

## 4. Defining Prognostic Immune Groups for New Immunotherapies

### 4.1. Overview of Immunotherapy in Urothelial Carcinoma

The approval of atezolizumab and pembrolizumab for platinum-refractory locally advanced/metastatic UC (la/mUC) in 2016–2017 marked the beginning of the ICI era in UC. Subsequent approvals extended across multiple settings: pembrolizumab as first-line monotherapy in cisplatin-ineligible PD-L1-high patients (KEYNOTE-052), avelumab as first-line maintenance following platinum stabilization (JAVELIN Bladder 100), nivolumab as adjuvant therapy after radical surgery in high-risk MIBC (CheckMate 274), and pembrolizumab as perioperative therapy (AMBASSADOR). Most recently, the phase III EV-302/KEYNOTE-A39 trial demonstrated that enfortumab vedotin plus pembrolizumab significantly improved overall survival compared to platinum-based chemotherapy in treatment-naïve la/mUC, establishing this combination as a new standard of care [2]. This shift toward first-line ADC–ICI combination therapy has important, but as yet largely unaddressed, implications for immune biomarker development. Biomarkers validated in the context of ICI monotherapy or ICI–chemotherapy combinations—including PD-L1 expression, TMB, and molecular subtype—were derived from trials in which clinical benefit was attributable, by design, to checkpoint blockade alone. In EV plus pembrolizumab, however, response results from two mechanistically distinct agents acting in parallel: an antibody–drug conjugate targeting Nectin-4, a cell-adhesion protein expressed largely independently of tumor immune infiltration, and an anti-PD-1 antibody whose activity is classically immune-phenotype-dependent. Whether existing immune biomarkers retain predictive value in this combination—and, if so, whether they predict benefit from the ICI component, the ADC component, or a synergistic interaction between the two—remains an open and clinically consequential question, discussed further in Section 4.5.

**Figure 2:**
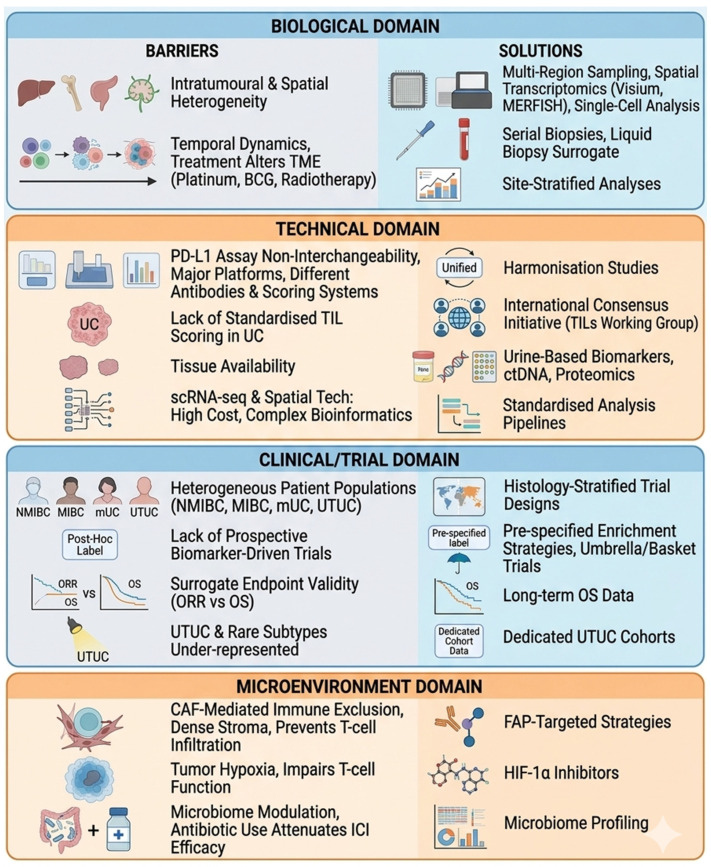
Schematic overview of the main barriers to studying the immune phenotype in urothelial carcinoma research, organized by biological, technical, clinical/trial and microenvironment domains, with their corresponding proposed solutions.

Despite this progress, ICI monotherapy response rates remain modest (15–25%), and predictive biomarkers capable of identifying the minority of long-term responders are urgently needed. The modest ICI response rates observed despite UC’s high mutational burden and historical BCG-responsiveness likely reflect additional, UC-specific determinants of immune escape—including chronic inflammatory exposures such as tobacco carcinogens and endemic schistosomiasis, and the unique urinary microbiome—that extend beyond the generic mechanisms discussed in this review and are summarized among the broader barriers depicted in Figure 2.

The complexity of the UC immune phenotype—heterogeneous, dynamic, and modulated by molecular subtype, prior therapies, and anatomical site—necessitates a multiparameter approach to biomarker development.

### 4.2. Established Predictive Biomarkers

Before reviewing individual biomarkers, it is important to distinguish between prognostic and predictive biomarkers, as this distinction is central to clinical translation and is not always made explicit in the UC biomarker literature. A prognostic biomarker provides information on the likely disease course or clinical outcome (e.g., overall survival, recurrence) largely independently of the treatment received, identifying patients with inherently more or less favorable disease biology. A predictive biomarker, in contrast, provides information on the likely relative benefit from a specific therapeutic intervention compared with another; its clinical utility depends on comparison between differently treated groups, ideally in a randomized setting. A biomarker may be purely prognostic, purely predictive, or both—for example, tumor-infiltrating lymphocyte (TIL) density is prognostic for survival largely irrespective of treatment [8,11], whereas the CD8+/FoxP3+ ratio additionally shows predictive value for response to immune checkpoint inhibition [19]. Throughout this section and in Table 2, each biomarker is explicitly classified according to this framework to clarify its intended clinical application.

It is important to note that the majority of the biomarker evidence discussed below derives from studies conducted in MIBC and metastatic UC cohorts treated with ICI; comparatively little validated biomarker data exists specifically for NMIBC populations treated with pembrolizumab (Section 3.4.1) or for UTUC, and extrapolation of these biomarkers to those settings should be made with caution.

#### 4.2.1. PD-L1 Expression

PD-L1 protein expression, assessed by immunohistochemistry (IHC), was the first approved predictive biomarker for ICI in UC and remains the most widely used in clinical practice, although—as discussed below—its clinical utility has been increasingly questioned. Four major assay platforms are employed: SP142 (atezolizumab), 22C3 (pembrolizumab), 28-8 (nivolumab), and SP263 (durvalumab). These differ in antibody selection, scoring methodologies (immune cell score [IC], tumor proportion score [TC], combined positive score [CPS]), and tissue preparation requirements [4]. This platform non-interchangeability represents a major clinical challenge: a patient may be PD-L1-positive by one assay and negative by another. To this issue must be added tumor heterogeneity, since the evaluation is performed on tissue sections rather than the whole sample, which may introduce errors in the assessment.

In UC, PD-L1 expression demonstrates modest and context-dependent predictive utility for ICI benefit. In first-line cisplatin-ineligible patients, PD-L1 expression (IC ≥ 5% for atezolizumab; CPS ≥ 10 for pembrolizumab) enriches for response and was used to guide approval labelling, although there is significant overlap in clinical outcomes among the subgroups defined based on PD-L1 [60]. In second line and maintenance settings, PD-L1 has limited additional predictive value beyond response to prior platinum therapy. The role of PD-L1 expression in guiding biomarker-directed treatment selection is increasingly being replaced by multiparameter approaches incorporating molecular subtype and additional immune markers [61]. Reflecting this limited and inconsistent performance, PD-L1 is increasingly regarded as a historically important but clinically limited biomarker rather than a cornerstone of biomarker-driven decision-making in UC, with most currently approved first-line regimens recommended irrespective of PD-L1 status [4,61].

Dynamic PD-L1 expression—fluctuating in response to IFN-γ signaling, treatment, and clonal selection—further complicates its use as a static biomarker. Promoter hypomethylation of the PD-L1 gene (CD274) has been proposed as an epigenetic predictor of ICI response that may capture tumors with inducible, rather than constitutive, PD-L1 expression [62].

#### 4.2.2. FGFR3 Alterations

FGFR3 mutations/fusions occur in ~20% of la/mUC (up to 70% of low-grade NMIBC) and are associated with the luminal papillary subtype and an immunosuppressive TME, marked by reduced CD8+ infiltration, impaired antigen presentation, and PD-L1 degradation via NEDD4 [40,41]. However, FGFR3 status should be regarded as a contextual, not absolute, negative biomarker for ICI benefit: in a real-world cohort, FGFR3-altered and wild-type tumors showed statistically equivalent ORR (12% vs. 19%; *p* = 0.73), with the CD8+/immunosuppressive signature balance—not FGFR3 status alone—driving response [40]. Its predictive relevance is further modulated by concurrent TMB [42], molecular subtype, PD-L1 expression, metastatic site, prior treatment, and the genomic assay platform used for testing.

Erdafitinib is approved for FGFR3/2-altered la/mUC (THOR trial) as a targeted alternative to ICI [63], and preclinical data show FGFR3 inhibition can resensitize tumors to anti-PD-1 by reversing T-cell exclusion [27], supporting combination strategies under investigation. Taken together, FGFR3 alteration alone is insufficient to exclude patients from ICI benefit; its clinical utility depends on integration with co-existing variables rather than use as a standalone rule-out criterion.

#### 4.2.3. Tumor Mutational Burden

TMB, defined as the number of somatic non-synonymous mutations per megabase of sequenced genome, correlates with neoantigen burden and CTL priming capacity. UC has among the highest TMB of all solid tumors (median approximately 8 mut/Mb), partially explaining its responsiveness to ICI as a class. Pembrolizumab received FDA approval for TMB-high (≥10 mut/Mb) solid tumors based on KEYNOTE-158, but the specific predictive value in UC is less clear-cut than in other tumor types [64].

In UC, the relationship between TMB and ICI benefit is complex: FGFR3-mutant tumors, despite having TMB in the intermediate range, tend to show comparatively lower ICI response rates in some but not all cohorts [42], whereas MSI-H tumors with very high TMB show excellent responses. Moreover, the type and context of mutations (clonal vs. subclonal, specific driver mutations) may be more relevant than raw TMB, and the predictive value of TMB appears additive rather than superior to PD-L1 and molecular subtype when combined in multivariate models [65].

#### 4.2.4. Mismatch Repair Deficiency and Microsatellite Instability

MMR deficiency (dMMR) and high microsatellite instability (MSI-H) occur in approximately 3–5% of la/mUC, with higher prevalence in UTUC (particularly Lynch syndrome-associated tumors). Pembrolizumab is approved for dMMR/MSI-H solid tumors regardless of origin, and UC patients with this phenotype show high response rates and durable remissions to ICI [66]. Importantly, MSI-H bladder and UTUC tumors display a highly inflamed immune phenotype with abundant CD8+ TIL, dense TLS, and elevated PD-L1 expression, consistent with a neoantigen-driven immune response [54]. Testing for dMMR/MSI-H should be considered in all la/mUC patients, particularly those with UTUC or a personal/family history suggestive of Lynch syndrome.

### 4.3. Emerging Immune Biomarkers

The biomarkers discussed in this section are, with few exceptions, derived from small, single-institution, retrospective cohorts that have not been validated in independent datasets; their relative evidence strength—none currently meeting criteria for prospective validation—is summarized explicitly in the Evidence Level column of Table 2, and findings should be interpreted as hypothesis-generating rather than clinically actionable until confirmed in larger, independent, and ideally prospective cohort.

#### 4.3.1. Tumor-Infiltrating Lymphocytes

TIL density, assessed by H&E or IHC on routine tumor sections, is the most clinically accessible immune biomarker in UC. Multiple studies have validated CD8+ TIL density as an independent prognostic factor for survival in resected MIBC [8,11]; this prognostic association was established prior to the ICI era and does not, by itself, constitute evidence of predictive value for ICI benefit specifically. The stromal TIL (sTIL) scoring methodology, analogous to the breast cancer TIL scoring guidelines, offers a reproducible and quantitative approach applicable to UC, though formal consensus guidelines have not yet been established for this tumor type [45].

Beyond total TIL counts, the functional state and spatial distribution of TILs refine prognostic stratification. The CD8/Foxp3 ratio—quantifying the balance between cytotoxic and regulatory T cells—outperforms CD8+ density alone in predicting both tumor recurrence and response to BCG immunotherapy in NMIBC [19]; however, its predictive value for ICI response in MIBC has not yet been formally validated and should not be assumed from NMIBC/BCG data. Immunoscore-based scoring of CD3+ and CD8+ T-cell densities at the invasive margin and tumor center adds independent prognostic value over TNM stage [10], although this finding needs prospective validation. Digital pathology and AI-assisted TIL quantification are emerging as scalable solutions to standardize TIL assessment across large cohorts, overcoming the inter-observer variability inherent to manual scoring [67].

#### 4.3.2. HLA Class I Expression

HLA class I (HLA-I) molecules are essential for CD8+ T-cell recognition of tumor-derived neoantigens and constitute a critical checkpoint in the cancer-immunity cycle. HLA-I loss or downregulation driven by somatic mutations, copy number variation, or epigenetic silencing represents one of the most common mechanisms of immune escape in UC, affecting approximately 40–60% of MIBC [45]. Tumors with complete HLA-I loss represent an extreme form of immune escape characterized by T-cell exclusion and ICI resistance, whereas tumors with retained HLA-I expression combined with negative PD-L1 define a distinct subgroup with immune rejection capacity and potentially superior ICI responsiveness [37].

HLA class II expression on tumor cells, induced by IFN-γ signaling, enables direct CD4+ T-cell recognition and has been associated with improved ICI outcomes in some solid tumors. In UC, HLA-II expression correlates with an inflamed immune phenotype and superior response to atezolizumab, particularly in PD-L1-low tumors [12]. Integrating HLA-I and HLA-II expression into immune phenotype scoring systems may improve predictive accuracy beyond PD-L1 alone.

#### 4.3.3. Tertiary Lymphoid Structures

TLS are ectopic lymphoid aggregates that form within tumors in response to chronic inflammation and antigen stimulation. Mature TLS contain germinal center-like structures with follicular helper T cells, B cells, and follicular dendritic cells, generating local adaptive immune responses. In MIBC, high TLS density is associated with improved survival, response to neoadjuvant chemotherapy, and ICI benefit [32,33]. CXCL13—a chemokine produced by Tfh cells and serving as a surrogate marker for TLS presence—is detectable in tumor tissue and plasma and has been associated with ICI response in multiple cohorts [34].

Induction of TLS formation has emerged as a therapeutic strategy to convert immunologically cold tumors into TLS-enriched hot tumors. Neoadjuvant chemoimmunotherapy promotes TLS formation in MIBC, and this effect correlates with pathological response [47]. Strategies to pharmacologically induce TLS—through TLR agonists, STING agonists, or cytokine-based approaches—are under active investigation in combination with ICI.

#### 4.3.4. Multiparameter Transcriptomic Immune Scores

Given the limitations of single biomarkers, multiparameter gene expression-based immune scores have been developed to capture the complexity of the UC immune phenotype. The T-cell-to-stroma enrichment (TSE) score integrates transcriptomic quantification of T-cell infiltration and stromal fibroblast content, predicting ICI response across multiple UC cohorts independently of molecular subtype [33]; however, further prospective validation is required. The 27-gene IO Score, a commercially available RNA-based assay, predicts ICI benefit in metastatic UC independently of FGFR3 status and PD-L1 expression [68]. The Immunotherapy Response Score (IRS) integrates clinical and molecular variables and has been validated in the ARON-2 datasets [69]. Clinical translation of the TSE score and similar transcriptomic assays remains limited by platform requirements, turnaround time, and cost relative to routine IHC, and cross-platform reproducibility has not been established [32,70]. Notably, IHC-based methods are not inherently simpler in practice: PD-L1 scoring already shows substantial inter-platform variability requiring standardized criteria [4,12,34,60,62], a limitation likely to extend to pathologist training needs for any newly incorporated assay.

The integration of immune gene expression profiles with single-cell and spatial data is generating increasingly refined immune phenotype classifications. A consensus taxonomy of UC immune phenotypes—analogous to the breast cancer PAM50 classification—would facilitate cross-trial comparison and enable biomarker-driven patient stratification in future ICI studies.

### 4.4. Towards a Multiparameter Immune Score in UC

The convergence of evidence from transcriptomic profiling, IHC, and clinical trial correlative studies argues compellingly for a multiparameter approach to immune biomarker development in UC. No single biomarker—PD-L1, TMB, FGFR3, TIL density, or molecular subtype—captures the full complexity of the immune microenvironment or achieves sufficient positive and negative predictive value for clinical use.

Proposed integrative biomarker frameworks for UC include the following: (1) a combined molecular subtype × immune phenotype (hot/cold/excluded/desert) classification as the primary patient stratification layer; (2) PD-L1 and HLA-I/II co-expression as mechanistic predictors of checkpoint blockade vulnerability; (3) TLS/CXCL13 as surrogate of adaptive immune activation [70]; (4) FGFR3 status as a contextual, subtype- and TMB-dependent modulator of ICI benefit rather than an absolute negative predictor, informing rational combination strategies; and (5) liquid biopsy (ctDNA, circulating immune cells) for dynamic response monitoring [25]. 

It is important to note that this framework is presented as a conceptual integration strategy rather than a prescriptive clinical algorithm: current evidence is insufficient to define specific, validated treatment-selection rules—for example, which precise phenotype–biomarker combination should direct ICI monotherapy versus combination therapy, or FGFR3-targeted therapy versus its combination with ICI. Prospective, biomarker-stratified trials are required before such algorithms can be responsibly proposed; the barriers to reaching this stage are discussed in detail in Section 5.

### 4.5. Biomarker Development in the Era of ADC–ICI Combinations

The establishment of enfortumab vedotin (EV) plus pembrolizumab as first-line standard of care for la/mUC [2] fundamentally reshapes the biomarker landscape described above. Nectin-4, the target of EV, is a cell-adhesion molecule of the immunoglobulin superfamily that is overexpressed in the large majority of urothelial carcinomas largely independently of molecular subtype and baseline immune infiltration [71,72,73]. Its expression is therefore not captured by any of the immune-centric biomarkers discussed in Section 4.2, Section 4.3 and Section 4.4 (PD-L1, TIL density, TLS, immune phenotype class), raising the possibility that a proportion of EV–pembrolizumab responders derive benefit predominantly from the ADC component, independently of baseline immune status.

Conversely, preclinical and early translational data indicate that MMAE-mediated cytotoxicity can induce immunogenic cell death (ICD)—characterized by calreticulin exposure, HMGB1 release, and ATP secretion—which in turn promotes dendritic cell maturation and recruitment of cytotoxic CD8+ T cells into previously immune-excluded or immune-desert tumors [74]. This ADC-induced immune remodeling (Section 3.4.3) provides a plausible mechanistic basis for synergy with checkpoint blockade but also implies that a tumor’s baseline immune phenotype may not predict its immune phenotype after ADC exposure—a critical caveat for any biomarker strategy based on pre-treatment biopsies alone.

These considerations converge on an unresolved and clinically important question: do the immune biomarkers described in this review predict benefit from the ICI component of EV–pembrolizumab, from the ADC component, or specifically from their combination? The pivotal EV-302/KEYNOTE-A39 trial did not report efficacy stratified by baseline PD-L1 expression, TIL density, or immune phenotype class [2], leaving this question unanswered at the population level. Addressing it will require the following: (1) correlative biomarker analyses of EV-302 and similar trials stratifying outcomes by baseline immune phenotype; (2) paired pre- and post-ADC biopsies to directly characterize treatment-induced immune remodeling, analogous to studies already performed for platinum chemotherapy and BCG (Section 4.1); and (3) mechanistic studies dissociating Nectin-4-dependent cytotoxicity from ICD-mediated immune activation. Until such data become available, immune biomarkers developed and validated in the ICI-monotherapy era should be applied to ADC–ICI combination regimens with caution, and their predictive value in this setting should be regarded as hypothesis-generating rather than established.

Table 2 lists the main prognostic and predictive biomarkers according to their level of clinical evidence and applicability.

**Table 2:** Established and emerging immune biomarkers in urothelial carcinoma: evidence level and clinical applicability.

Biomarker	Type	Clinical Role	Prognostic/ Predictive	Key Limitations	Evidence Level	References
PD-L1 (SP142/22C3/28-8/SP263)	Protein IHC	Predictive: 1L cisplatin-ineligible UC (atezolizumab IC ≥ 5%; pembrolizumab CPS ≥ 10)	Predictive	Platform non-interchangeability; intratumoral heterogeneity; dynamic expression	Approved (companion diagnostic)	[4,12,34,60,62]
FGFR3 mutation/fusion	DNA/RNA	Predictive: response to erdafitinib; contextual negative predictor for ICI (cold TME)	Predictive (dual-directional)	Context-dependent effect (TMB, subtype, assay platform); secondary resistance; co-mutations	Approved (companion diagnostic)	[27,40,41,42,63]
TMB (≥10 mut/Mb)	WES/panel NGS	Pan-tumor ICI predictor; modest in UC alone	Predictive	Threshold variability; assay differences; not UC-specific	Approved (FDA, pan-tumor)	[64,65]
MMR deficiency/MSI-H	IHC/PCR/NGS	Predictive: pembrolizumab (pan-tumor); UC prevalence ~3–5%	Predictive	Low prevalence in UC; incomplete overlap dMMR/MSI-H	Approved (indication)	[54,64,66]
TIL density (CD8+/Foxp3+)	IHC/H&E	Prognostic; CD8/Foxp3 ratio predictive of BCG response (NMIBC)	Both	Scoring non-standardized; intratumor heterogeneity; no cut-off consensus	Retrospective evidence (prognostic validation studies)	[8,9,10,11,17,19,44]
HLA class I expression	IHC/WES	Immune escape biomarker; HLA-I loss correlates with T-cell exclusion and ICI resistance	Predictive	Complex genetics; LOH assessment technically demanding	Exploratory (mechanistic studies)	[30,37,45]
Tertiary lymphoid structures (TLSs)	IHC/gene signature	Prognostic in MIBC; surrogate: CXCL13 expression	Prognostic	Evaluation not standardised; no prospective ICI data	Retrospective evidence (cohort studies)	[31,32,34,70]
Immune phenotype class (hot/cold/excluded/desert)	Transcriptomic/IHC	Integrative prognostic/predictive framework; correlates with ICI benefit	Both	Requires bulk or single-cell transcriptomics; not routinely available	Retrospective evidence; needs prospective validation	[6,12,13,22,33,35,37]
27-gene IO score/TSE score	Gene expression	Predictive of ICI benefit independent of FGFR status	Predictive	Commercial platforms; limited external validation	Retrospective evidence (single cohort)	[33,68,69]
ctDNA dynamics	Liquid biopsy	Early response assessment; resistance and monitoring	Predictive (dynamic)	No standardised assay; timing unclear	Exploratory	[25,34,75,76]

Evidence Level categories: Approved (regulatory-approved companion diagnostic or indication); Prospectively validated (confirmed in a prospective biomarker-driven trial); Retrospective evidence (retrospective cohort or single-cohort studies); Exploratory (mechanistic or hypothesis-generating studies). No biomarker in this table currently meets criteria for the Prospectively validated category. CPS, combined positive score; ctDNA, circulating tumor DNA; dMMR, mismatch repair deficiency; FGFR3, fibroblast growth factor receptor 3; IC, immune cell score; ICI, immune checkpoint inhibitor; IHC, immunohistochemistry; IRS, immunotherapy response score; MSI-H, high microsatellite instability; NGS, next-generation sequencing; PD-L1, programmed death-ligand 1; TC, tumor cell score; TIL, tumor-infiltrating lymphocyte; TLS, tertiary lymphoid structures; TMB, tumor mutational burden; TME, tumor microenvironment; TSE, T-cell-to-stroma enrichment score; UC, urothelial carcinoma; WES, whole-exome sequencing.

## 5. Main Barriers to Studying the Immune Phenotype in Urothelial Carcinoma

### 5.1. Biological Barriers

The most fundamental challenge in UC immune phenotype research is the pronounced spatial and temporal heterogeneity of the TME. Immune infiltration varies markedly between tumor regions, with differential abundance of CD8+ T cells, TAMs, and MDSCs at the tumor center, invasive margin, and stroma [17]. Paired sampling of primary and metastatic lesions reveals substantial divergence in immune phenotype between sites, and the metastatic TME—more clinically relevant for ICI response prediction—is rarely captured in routine clinical practice [50].

Treatment-induced remodeling of the TME constitutes an additional biological complication. Prior platinum chemotherapy, radiotherapy, and BCG all modify the immune landscape in ways that confound baseline immune phenotype assessment. Neoadjuvant platinum increases CD8+ TIL density and PD-L1 expression [28], BCG induces local Treg expansion [19], and radiotherapy can both activate and suppress immune responses depending on dose and fractionation [47]. Biomarker studies that fail to account for treatment history may generate inconsistent or misleading conclusions.

The site-specific determinants of immune microenvironment—including the unique immunological milieu of the liver, bone marrow, and lymphatic tissue—create organ-specific TME profiles in metastatic UC that differ systematically from the primary tumor and from each other [49]. Understanding and accounting for these site-specific differences is essential for the development of clinically meaningful biomarker strategies.

A further, often underappreciated limitation of immune-phenotype-centric frameworks is that tumor cell-intrinsic resistance mechanisms—including antigen presentation machinery defects beyond HLA-I loss (Section 4.3.2), interferon-gamma signaling pathway mutations (e.g., JAK1/JAK2), and epigenetic silencing of neoantigen expression—can confer ICI resistance independently of, and can override, a favorable immune infiltrate phenotype. A densely infiltrated, “hot” tumor is therefore not a guarantee of ICI responsiveness if downstream antigen presentation or interferon signaling is disrupted, a limitation not captured by immune cell quantification alone [37,45].

### 5.2. Technical and Methodological Barriers

The non-interchangeability of PD-L1 assay platforms remains the most clinically consequential technical barrier in UC immunotherapy. The four approved assays use different antibodies (SP142, 22C3, 28-8, SP263), different scoring systems (IC, TC, CPS), and different cut-off values, generating discordant results for up to 30% of patients when platforms are compared directly [4]. Harmonization efforts and transcriptomic surrogates for PD-L1 expression may partially address this, but prospective validation in registrational trials is required before alternative approaches can replace companion diagnostics.

The absence of standardized TIL scoring guidelines for UC—in contrast to breast cancer, which has well-validated H&E-based sTIL scoring recommendations—represents a significant barrier to cross-study comparability. Variations in tissue processing (formalin fixation, section thickness, staining protocols), scoring methodology (manual vs. digital), and cut-off definition generate inter-laboratory variability that limits the reproducibility of TIL-based biomarker studies [45].

Tissue availability poses a particular challenge in UTUC, where small ureteroscopic biopsies provide limited material for multi-analyte biomarker testing. Similarly, decalcified bone metastasis specimens, FFPE material from transurethral resection, and liquid-preserved cytology samples are suboptimal for many molecular and immunological assays. Urine-based biomarkers—including urinary cell-free DNA, urinary cytokines, and urine single-cell transcriptomics—offer a non-invasive alternative that deserves further development [75].

Advanced technologies such as scRNA-seq, spatial transcriptomics, and mass cytometry provide unparalleled resolution but remain expensive, technically demanding, and bioinformatically complex. The lack of standardized analysis pipelines, reference atlases specific to UC, and validated computational tools for clinical application limits the translation of these discoveries into actionable biomarkers [6]. These platforms are further constrained by well-recognized technical artifacts—dropout events causing false-negative gene detection, batch effects across sequencing runs, and, for most spatial platforms, panel-limited rather than whole-transcriptome gene coverage [6]. At present, these technologies retain substantial value for mechanistic discovery but remain distant from routine clinical incorporation given these combined technical, financial, and translational barriers.

### 5.3. Clinical and Trial Design Barriers

Patient population heterogeneity in ICI trials—encompassing NMIBC, MIBC, la/mUC, bladder and UTUC, pure urothelial and variant histologies—is a key source of signal dilution that hinders the detection of specific immune phenotype subgroups. Variant histologies and UTUC are systematically underrepresented or excluded from major trials, generating evidence gaps in precisely the populations where biology may most diverge from conventional UC [54].

Biomarker analyses in UC-ICI trials have predominantly been post hoc, exploratory, and conducted on small, unrepresentative tissue subsets. The absence of pre-specified, analytically validated biomarker endpoints with appropriate statistical power has limited the regulatory and clinical utility of trial correlatives. Prospective biomarker-driven trial designs—with pre-specified enrichment strategies, central testing, and formal decision-making algorithms—are urgently needed to establish the clinical utility of emerging immune biomarkers in UC.

Regulatory and reimbursement policies for new companion diagnostics in UC are behind current biological evidence. The validation pathway for multiparameter immune scores, digital pathology-based TIL quantification, and spatial transcriptomic classifiers in oncology remains undefined, creating barriers to clinical implementation even when analytical validity has been established. 

Two obstacles recur throughout this review and merit explicit translation into trial design requirements: spatial heterogeneity and temporal heterogeneity. Addressing spatial heterogeneity requires that biomarker-driven trials mandate multi-region tumor sampling (or whole-slide/spatial profiling rather than single-core biopsies) [17], pre-specify how discordant regional results will be reconciled into a single reportable biomarker call, and, for metastatic disease, prioritize biopsy of the treatment-relevant lesion over archival primary tissue [49,50]. Addressing temporal heterogeneity requires paired pre- and on-treatment (or pre- and post-ADC) biopsies as a mandatory correlative endpoint in ADC-ICI combination trials [74], standardized biopsy timing relative to treatment cycles, and explicit reporting of prior treatment exposure when interpreting any biomarker result [19,28,47]. Without these design requirements, biomarker-outcome associations reported in the current literature remain vulnerable to sampling artifact and cannot be assumed to generalize to a patient’s tumor at the time of treatment decision-making.

### 5.4. The Tumor Microenvironment Beyond Immune Cells

The non-immune compartment of the UC TME—including CAFs, endothelial cells, and the extracellular matrix—plays a critical modulatory role that has received less attention than immune cells but is equally important for therapeutic response.

CAFs in UC produce TGF-β, VEGF, CXCL12, and other factors that create a physical and chemical barrier to T-cell infiltration, promote M2 macrophage polarization, and recruit MDSCs [44]. The stroma-rich molecular subtype, characterized by dominant CAF signatures, exemplifies the immune-excluded phenotype in which T cells accumulate at the tumor periphery but fail to penetrate the CAF-rich core. FAP-targeted strategies and TGF-β blockade combined with ICI have shown activity in preclinical UC models and are being evaluated clinically. Beyond CAF-driven stromal exclusion, abnormal tumor vasculature, pericyte dysfunction, and endothelial anergy—impairing T-cell adhesion and transendothelial migration independently of hypoxic signaling—represent additional, mechanistically distinct barriers to immune infiltration, likely requiring vascular-normalizing rather than stromal-targeted therapeutic strategies [77].

Tumor hypoxia, driven by abnormal vasculature and high metabolic demand in MIBC, upregulates HIF-1α, promotes PD-L1 expression on tumor cells and TAMs, impairs CD8+ T-cell function, and facilitates MDSC accumulation [72]. Hypoxia-informed biomarker strategies and HIF-targeted combinations with ICI represent rational approaches to addressing this barrier.

The gut and bladder microbiome modulate ICI efficacy through multiple mechanisms, including effects on systemic immune tone, local innate immune activation, and direct metabolite-mediated immunomodulation. Antibiotic use at the time of ICI initiation is associated with reduced ICI efficacy in UC across multiple retrospective cohorts, emphasizing the clinical relevance of microbiome–immune interactions and arguing for prospective microbiome characterization in ICI trials [78] (Table 3; Figure 2).

## 6. Future Directions

### 6.1. Novel Immune Checkpoints and Combination Strategies

Beyond the PD-1/PD-L1 axis, multiple co-inhibitory receptors expressed on TILs in UC have been identified as therapeutic targets. LAG-3 (lymphocyte-activation gene 3), TIM-3 (T-cell immunoglobulin and mucin domain 3), TIGIT (T-cell immunoreceptor with Ig and ITIM domains), VISTA, and Siglec-15 are expressed on tumor-infiltrating CD8+ T cells in UC and contribute to T-cell exhaustion and ICI resistance [14,45]. LAG-3 expression on peripheral blood lymphocytes has been associated with poorer outcomes following ICI in UC, suggesting a role as a dynamic on-treatment biomarker [45]. Co-blockade of PD-1 and TIGIT has shown pre-clinical synergy in bladder cancer through reinvigoration of terminally exhausted TOX+ CD8+ T cells, and phase II/III trials are underway in solid tumors [14].

Converting immunologically cold and excluded tumors to the hot phenotype—‘immune activation’—represents a compelling therapeutic paradigm. Strategies include: FGFR3 inhibition to relieve T-cell exclusion [27]; DNA-demethylating agents (guadecitabine) to induce PD-L1 expression and neoantigen presentation; epigenetic modulation targeting HDAC7 to restore CTL infiltration [79]; STING agonists to activate innate-adaptive immune crosstalk; oncolytic viruses to generate immunogenic cell death and TLS induction; and radiotherapy combinations using the abscopal effect [72].

It should be noted that clinical results for these agents across oncology have been mixed—LAG-3 blockade is now approved in melanoma, whereas the TIGIT inhibitor tiragolumab failed its primary endpoint in a phase III lung cancer trial—and that UC-specific late-phase clinical data remain immature, with current evidence derived largely from expression-profiling and ex vivo reinvigoration studies rather than completed efficacy trials. These targets should therefore be regarded as an active but still early-stage area of investigation in UC specifically.

### 6.2. Artificial Intelligence and Digital Pathology

AI-assisted digital pathology is transforming immune phenotype assessment in solid tumors. Deep learning models trained on H&E and multiplex IHC images can quantify TIL density, classify immune phenotype (hot/cold/excluded/desert), identify TLS, and predict molecular subtype and ICI response from routine pathological material with accuracy approaching expert human performance [67]. AI-informed computational pathology classifiers have already demonstrated predictive value for ICI outcomes across treatment modalities in MIBC, offering a scalable and cost-effective approach to immune phenotype-guided therapy selection [67].

The integration of AI-based pathology with multi-omics data—integrating genomic, transcriptomic, epigenomic, and proteomic layers—will generate composite biomarker profiles of unprecedented granularity. Cloud-based pathology platforms enabling central AI-assisted analysis of digitized slides from global clinical trials will facilitate the large-scale, prospective biomarker studies needed to validate and implement these tools in routine clinical practice.

Realizing this potential in clinical practice, however, requires more than analytical validation. Concrete implementation would require: (1) prospective, multi-center studies in which AI-derived immune phenotype calls are generated in real time and used to guide treatment allocation, rather than applied retrospectively to archival cohorts [67]; (2) regulatory-grade lock-down of algorithms prior to trial use, with pre-specified performance thresholds; and (3) integration into pathology reporting workflows compatible with existing turnaround-time constraints in genitourinary oncology. 

It is important to emphasize that this field remains at a very early stage of development: virtually all studies cited above are retrospective, single-institution, and have not undergone prospective validation, and substantial additional hurdles—including regulatory approval pathways specific to AI-based diagnostics, reimbursement mechanisms, and formal pathologist training and accreditation programs for AI-assisted interpretation—remain largely unaddressed and will each require dedicated development before clinical adoption is feasible.

A persistent and underacknowledged bottleneck is the disconnect between spatial omics data and clinical outcomes: most spatial transcriptomic and multiplex imaging studies to date are small, single-institution, retrospective, and correlate spatial architecture with survival post hoc, without prospective validation of any single spatial metric as a decision-making tool [17,18,21]. Bridging this gap will require spatial biomarkers to be prospectively tested within the same trial-design framework outlined in Section 5.3, rather than treated as an inherently superior technology whose clinical utility can be assumed.

### 6.3. Liquid Biopsy and Dynamic Immune Monitoring

Liquid biopsy offers a minimally invasive approach to longitudinal immune phenotype monitoring during ICI therapy. Circulating tumor DNA (ctDNA) kinetics under ICI treatment have been associated with response and resistance evolution in la/mUC [76]; however, ctDNA clearance primarily reflects tumor cell death rather than immune activity per se, and its interpretation as a real-time immune readout is complicated by reports of delayed clearance in some responders and transient ctDNA increases during pseudoprogression, a pattern conceptually analogous to the inflammatory flare observed with other blood-based markers [34,76]. The integration of ctDNA with circulating immune cell phenotyping—including changes in peripheral blood NK cells, MDSC frequencies, and T-cell clonality—may provide real-time readouts of immune response that complement tissue-based biomarkers. Urine-based biomarkers, including urinary cytokines, cell-free RNA, and exosomal cargo, offer a non-invasive and tumor-proximal sampling strategy particularly well-suited to bladder UC surveillance and response monitoring [75].

## 7. Conclusions

The immune microenvironment of urothelial carcinoma is a complex, dynamic, and biologically rich ecosystem that fundamentally determines disease outcome and therapeutic response. The field has progressed from the early recognition that BCG immunotherapy requires an intact adaptive immune response, through the identification of molecular subtypes with distinct immune contextures, to the current era of high-resolution single-cell and spatial profiling revealing the full diversity of the UC immune landscape. Rather than a fixed biomarker to be measured once, the immune phenotype of urothelial carcinoma is best understood as a dynamic clinical variable, continuously reshaped by four convergent forces traced throughout this review. First, intrinsic molecular programming establishes a baseline immune contexture: basal/squamous tumors are constitutively inflamed, while luminal papillary and neuronal tumors default to an immune-cold state anchored in FGFR3 signaling and neuroendocrine differentiation, respectively. Second, this baseline is not fixed in space or time: the immune phenotype evolves across disease stage, differs by anatomical and metastatic site, and diverges further in UTUC and variant histologies, which cannot be safely extrapolated from bladder UC alone. Third, immune phenotype is actively remodeled by treatment itself—BCG, platinum chemotherapy, radiotherapy, and, increasingly, antibody–drug conjugates, whose capacity to convert cold tumors into inflamed ones is reshaping how first-line ADC-ICI combinations should be biomarker-selected. Fourth, translating this complexity into clinical decisions remains constrained by a persistent actionability gap, rooted specifically in unresolved spatial heterogeneity [17,49,50] and temporal heterogeneity [19,28,47]: no single biomarker captures the UC immune phenotype in full and biological insight continues to outpace assay standardization and prospective validation.

Closing this gap—rather than further cataloguing biomarkers—should be the field’s organizing priority. The convergence of digital pathology, single-cell and spatial genomics, and AI-based analytics offers a realistic path to operationalizing immune phenotype as a serially measurable, treatment-responsive variable, guiding not only patient selection but also the sequencing of ADCs, checkpoint inhibitors, and emerging immunomodulatory strategies. The UC immune phenotype is not merely a scientific curiosity; it is the biological substrate upon which the next generation of therapeutic advances will be built.

## Figures and Tables

**Table 1:** Molecular subtypes of urothelial carcinoma and their associated immune phenotype characteristics.

Molecular Subtype	Immune Phenotype	Key TIL Features	PD-L1 Expression	ICI Response
Luminal papillary (TCGA LumP/Lund Urobasal A)	Cold/Immune desert	Low CD8+ TIL; depleted T-cell contexture; FGFR3-altered predominant	Low (IC0/TC0)	Lower
Luminal unstable (TCGA LumU)	Intermediate/Excluded	Moderate stromal TIL; limited intratumoral infiltration; DNA repair alterations	Variable	Intermediate
Luminal non-specified (Lund Urobasal B)	Immune-excluded	Stromal TIL present but unable to infiltrate tumor nest; TGF-β dominant	Low–intermediate	Lower–intermediate
Basal/squamous (TCGA BS/Lund SCC-like)	Hot/ Inflamed	High CD8+ TIL; high Treg; active immune response; CD8/Foxp3 imbalance	High (IC2/3)	Higher
Neuronal/ NE-like	Cold/Immune desert	Virtually absent TIL; strong neuroendocrine programme; immunosuppressive	Negative	Lower (uncertain)
Stroma-rich (Lund Mesenchymal-like)	Immune-excluded (fibroblast-driven)	Dense CAF stroma creates physical barrier; moderate TIL; EMT features	Low–moderate	Lower

ICI Response reflects relative, cohort-level associations rather than individual-patient certainty (Lower/Intermediate/Higher/uncertain); it is not a guarantee of individual treatment outcome. CAF, cancer-associated fibroblast; CD44, cluster of differentiation 44; EMT, epithelial-to-mesenchymal transition; FGFR3, fibroblast growth factor receptor 3; IC, immune cell score; ICI, immune checkpoint inhibitor; KRT5/6, keratin 5/6; KRT14, keratin 14; NE, neuroendocrine; PD-L1, programmed death-ligand 1; TC, tumor cell score; TCGA BS, The Cancer Genome Atlas basal/squamous subtype; TCGA LumP, The Cancer Genome Atlas luminal papillary subtype; TCGA LumU, The Cancer Genome Atlas luminal unstable subtype; TGF-β, transforming growth factor beta; TIL, tumor-infiltrating lymphocyte; Treg, regulatory T cell.

**Table 3:** Main barriers to studying the immune phenotype in urothelial carcinoma and proposed solutions.

Domain	Barrier	Proposed Solution/Future Direction
Biological	Intratumoral and spatial heterogeneity: immune phenotype varies across tumor regions and metastatic sites	Multi-region sampling; spatial transcriptomics (Visium, MERFISH); single-cell analysis of paired primary-metastasis
	Temporal dynamics: treatment alters TME (platinum, BCG, radiotherapy before sampling)	Serial biopsies; on-treatment sampling protocols; liquid biopsy as surrogate
	Site-specific TME differences: liver, bone, and nodal metastases have distinct immune landscapes	Site-stratified analyses in clinical trials; organotropism studies
Technical	PD-L1 assay non-interchangeability: four major platforms with different antibodies and scoring systems	Harmonization studies; transcriptomic PD-L1 surrogates; integrated scoring
	Lack of standardized TIL scoring in UC: no equivalent to breast cancer guidelines	International consensus initiative (analogous to TILs Working Group); digital pathology quantification
	Tissue availability: UTUC biopsies, small transurethral samples, decalcified bone metastases	Urine-based biomarkers; ctDNA; circulating immune cells; plasma proteomics
	scRNA-seq and spatial technologies: high cost, complex bioinformatics, not routine	Standardized analysis pipelines; open-access datasets; AI-assisted spatial phenotyping
Clinical/Trial	Heterogeneous patient populations: NMIBC, MIBC, mUC, UTUC, variant histologies mixed or excluded	Histology-stratified and disease-stage-specific trial designs
	Lack of prospective biomarker-driven trials in UC: most evidence from post hoc analyses	Pre-specified biomarker enrichment strategies; umbrella/basket platform trials
	Surrogate endpoint validity: ORR as surrogate for OS uncertain in ICI-treated UC	Long-term OS data; patient-level meta-analyses; milestone survival analyses
	UTUC and rare subtypes under-represented in clinical studies	Dedicated UTUC cohorts and substudies; mandatory variant histology reporting in trials
Micro- environment	CAF-mediated immune exclusion: dense stroma physically prevents T-cell infiltration	FAP-targeted strategies; TGF-β/VEGF blockade combination with ICI
	Tumor hypoxia: induces PD-L1 upregulation and impairs effector T-cell function	HIF-1α inhibitors in combination; hypoxia imaging as biomarker
	Microbiome modulation: antibiotic use attenuates ICI efficacy in UC	Microbiome profiling; probiotic/fecal microbiota transplant trials; antibiotic avoidance protocols

BCG, Bacillus Calmette–Guérin; CAF, cancer-associated fibroblast; ctDNA, circulating tumor DNA; FGFR3, fibroblast growth factor receptor 3; FFPE, formalin-fixed paraffin-embedded; ICI, immune checkpoint inhibitor; MERFISH, multiplexed error-robust fluorescence in situ hybridization; MIBC, muscle-invasive bladder cancer; NMIBC, non-muscle-invasive bladder cancer; ORR, objective response rate; OS, overall survival; PD-L1, programmed death-ligand 1; scRNA-seq, single-cell RNA sequencing; TIL, tumor-infiltrating lymphocyte; TLS, tertiary lymphoid structures; TLR, Toll-like receptor; TME, tumor microenvironment; UC, urothelial carcinoma; UTUC, upper tract urothelial carcinoma.

## Data Availability

No new data were created or analyzed in this study. Data sharing is not applicable to this article.

## Abbreviations

The following abbreviations are used in this manuscript:

ADC	Antibody–drug conjugate
BCG	Bacillus Calmette–Guérin
CAF	Cancer-associated fibroblast
CPS	Combined positive score
CTL	Cytotoxic T lymphocyte
ctDNA	Circulating tumor DNA
DC	Dendritic cell
dMMR	Mismatch repair deficiency
FGFR3	Fibroblast growth factor receptor 3
HLA	Human leucocyte antigen
IC	Immune cell score
ICI	Immune checkpoint inhibitor
IHC	Immunohistochemistry
la/mUC	Locally advanced or metastatic urothelial carcinoma
MDSC	Myeloid-derived suppressor cell
MIBC	Muscle-invasive bladder cancer
MMR	Mismatch repair
MSI-H	High microsatellite instability
NK	Natural killer (cell)
NMIBC	Non-muscle-invasive bladder cancer
ORR	Objective response rate
OS	Overall survival
pCR	Pathological complete response
PD-1	Programmed death-1
PD-L1	Programmed death-ligand 1
scRNA-seq	Single-cell RNA sequencing
TAM	Tumor-associated macrophage
TC	Tumor cell score
TCGA	The Cancer Genome Atlas
TIL	Tumor-infiltrating lymphocyte
TLS	Tertiary lymphoid structure
TMB	Tumor mutational burden
TME	Tumor microenvironment
Treg	Regulatory T cell
TSE	T-cell-to-stroma enrichment score

## References

[1] Sung H., Ferlay J., Siegel R.L., Laversanne M., Soerjomataram I., Jemal A., Bray F. (2021). Global Cancer Statistics 2020: GLOBOCAN Estimates of Incidence and Mortality Worldwide for 36 Cancers in 185 Countries. CA Cancer J. Clin..

[2] Powles T., Valderrama B.P., Gupta S., Bedke J., Kikuchi E., Hoffman-Censits J., Iyer G., Vulsteke C., Wheater M., Morales-Barrera R. (2024). Enfortumab Vedotin and Pembrolizumab in Untreated Advanced Urothelial Cancer. N. Engl. J. Med..

[3] Bellmunt J., de Wit R., Vaughn D.J., Fradet Y., Lee J.L., Fong L., Vogelzang N.J., Climent M.A., Petrylak D.P., Choueiri T.K. (2017). Pembrolizumab as Second-Line Therapy for Advanced Urothelial Carcinoma. N. Engl. J. Med..

[4] Maiorano B.A., Di Maio M., Cerbone L., Maiello E., Procopio G., Roviello G. (2024). Significance of PD-L1 in Metastatic Urothelial Carcinoma Treated with Immune Checkpoint Inhibitors: A Systematic Review and Meta-Analysis. JAMA Netw. Open.

[5] Robertson A.G., Kim J., Al-Ahmadie H., Bellmunt J., Guo G., Cherniack A.D., Hinoue T., Laird P.W., Hoadley K.A., Akbani R. (2017). Comprehensive Molecular Characterization of Muscle-Invasive Bladder Cancer. Cell.

[6] Liang Y., Tan Y., Guan B., Guo B., Xia M., Li J., Shi Y., Yu Z., Zhang Q., Liu D. (2022). Single-cell atlases link macrophages and CD8+ T-cell subpopulations to disease progression and immunotherapy response in urothelial carcinoma. Theranostics.

[7] Morales A., Eidinger D., Bruce A.W. (1976). Intracavitary Bacillus Calmette-Guérin in the treatment of superficial bladder tumors. J. Urol..

[8] Sharma P., Shen Y., Wen S., Yamada S., Jungbluth A.A., Gnjatic S., Bajorin D.F., Reuter V.E., Herr H., Old L.J. (2007). CD8 Tumor-Infiltrating Lymphocytes Are Predictive of Survival in Muscle-Invasive Urothelial Carcinoma. Proc. Natl. Acad. Sci. USA.

[9] Rouanne M., Betari R., Radulescu C., Goubar A., Signolle N., Neuzillet Y., Allory Y., Marabelle A., Adam J., Lebret T. (2019). Stromal lymphocyte infiltration is associated with tumour invasion depth but is not prognostic in high-grade T1 bladder cancer. Eur. J. Cancer.

[10] Li X.D., Huang C.W., Liu Z.F., Jiang L.J., Chen J.W., Xie D., Zhou F.J., Lu H.M., Liu Z.W. (2019). Prognostic Role of the Immunoscore for Patients with Urothelial Carcinoma of the Bladder Who Underwent Radical Cystectomy. Ann. Surg. Oncol..

[11] Kassouf W., Black P.C. (2020). Prognostic role of immunoscore in urothelial carcinoma of the bladder after radical cystectomy. Eur. Urol..

[12] Hamidi H., Senbabaoglu Y., Beig N., Roels J., Manuel C., Guan X., Koeppen H., Assaf Z.J., Nabet B.Y., Waddell A. (2024). Molecular heterogeneity in urothelial carcinoma and determinants of clinical benefit to PD-L1 blockade. Cancer Cell.

[13] Wang L., Sfakianos J.P., Beaumont K.G., Akturk G., Horowitz A., Sebra R.P., Farkas A.M., Gnjatic S., Hake A., Izadmehr S. (2021). Myeloid Cell-associated Resistance to PD-1/PD-L1 Blockade in Urothelial Cancer Revealed Through Bulk and Single-cell RNA Sequencing. Clin. Cancer Res..

[14] Han H.S., Jeong S., Kim H., Kim H.D., Kim A.R., Kwon M., Park S.H., Woo C.G., Kim H.K., Lee K.H. (2021). TOX-expressing terminally exhausted tumor-infiltrating CD8+ T cells are reinvigorated by co-blockade of PD-1 and TIGIT in bladder cancer. Cancer Lett..

[15] Desgrandchamps F., LeMaoult J., Goujon A., Riviere A., Rivero-Juarez A., Djouadou M., de Gouvello A., Dumont C., Wu C.-L., Culine S. (2018). Prediction of non-muscle-invasive bladder cancer recurrence by measurement of checkpoint HLA-G’s receptor ILT2 on peripheral CD8+ T cells. Oncotarget.

[16] Kim K., Alam S.M., Kuo F., Chen Z., Yip W., Katims A.B., Chu C., Lenis A.T., Hu W., Gokturk Ozcan G. (2025). Molecular Heterogeneity and Immune Infiltration Drive Clinical Outcomes in Upper Tract Urothelial Carcinoma. Eur. Urol..

[17] Debatin N.F., Bady E., Mandelkow T., Huang Z., Lurati M.C.J., Raedler J.B., Müller J.H., Vettorazzi E., Plage H., Samtleben H. (2024). Prognostic Impact and Spatial Interplay of Immune Cells in Urothelial Cancer. Eur. Urol..

[18] van Wilpe S., Gorris M.A.J., van der Woude L.L., Sultan S., Koornstra R.H.T., van der Heijden A.G., Gerritsen W.R., Simons M., de Vries I.J.M., Mehra N. (2022). Spatial and Temporal Heterogeneity of Tumor-Infiltrating Lymphocytes in Advanced Urothelial Cancer. Front. Immunol..

[19] Lim C.J., Nguyen P.H.D., Wasser M., Kumar P., Lee Y.H., Nasir N.J.M., Chua C., Lai L., Hazirah S.N., Loh J.J.H. (2021). Immunological Hallmarks for Clinical Response to BCG in Bladder Cancer. Front. Immunol..

[20] Kamatani T., Umeda K., Iwasawa T., Miya F., Matsumoto K., Mikami S., Hara K., Shimoda M., Suzuki Y., Nishino J. (2025). Clonal diversity shapes the tumor microenvironment leading to distinct immunotherapy responses in metastatic urothelial carcinoma. Nat. Commun..

[21] Gil-Jimenez A., van Dijk N., Vos J.L., Lubeck Y., van Montfoort M.L., Peters D., Hooijberg E., Broeks A., Zuur C.L., van Rhijn B.W.G. (2024). Spatial relationships in the urothelial and head and neck tumor microenvironment predict response to combination immune checkpoint inhibitors. Nat. Commun..

[22] Zhang Q., Zhang W., Lin T., Lu W., He X., Ding Y., Chen W., Diao W., Ding M., Shen P. (2022). Mass cytometry reveals immune atlas of urothelial carcinoma. BMC Cancer.

[23] Yoshida T., Ohe C., Ito K., Takada H., Saito R., Kita Y., Sano T., Tsuta K., Kinoshita H., Kitamura H. (2022). Clinical and molecular correlates of response to immune checkpoint blockade in urothelial carcinoma with liver metastasis. Cancer Immunol. Immunother..

[24] Sun X., Park M.D., Merad M., Brown B.D. (2026). Macrophages: Targets for next-generation cancer immunotherapy. Cancer Cell.

[25] Vanguri R.S., Smithy J.W., Li Y., Zhuang M., Maher C.A., Aleynick N., Peng X., Al-Ahmadie H., Funt S.A., Rosenberg J.E. (2023). Integration of peripheral blood- and tissue-based biomarkers of response to immune checkpoint blockade in urothelial carcinoma. J. Pathol..

[26] Széles Á., Kubik A., Váncsa S., Grünwald V., Hadaschik B., Ács N., Hegyi P., Nyirády P., Szarvas T. (2025). Prognostic and predictive value of pre-treatment blood-based inflammatory biomarkers in patients with urothelial carcinoma treated with immune checkpoint inhibitors. Front. Immunol..

[27] Okato A., Utsumi T., Ranieri M., Zheng X., Zhou M., Pereira L.D., Chen T., Kita Y., Wu D., Hyun H. (2024). FGFR inhibition augments anti-PD-1 efficacy in murine FGFR3-mutant bladder cancer by abrogating immunosuppression. J. Clin. Investig..

[28] Einerhand S.M.H., van Dijk N., van Dorp J., de Feijter J.M., van Montfoort M.L., van de Kamp M.W., Schaake E.E., Boellaard T.N., Hendricksen K., van der Heijden M.S. (2022). Survival after neoadjuvant/induction combination immunotherapy vs. combination platinum-based chemotherapy for locally advanced (Stage III) urothelial cancer. Int. J. Cancer.

[29] Guillamón C.F., Martínez-Sánchez M.V., Gimeno L., Mrowiec A., Martínez-García J., Server-Pastor G., Martínez-Escribano J.A., Torroba A., Ferri B., Abellán D.J. (2018). NK Cell Education in Tumor Immune Surveillance: DNAM-1/KIR Receptor Ratios as Predictive Biomarkers for Solid Tumor Outcome. Cancer Immunol. Res..

[30] Zhang L., Liu Z., Ding Y., Sun J., Wu Y., Su X., Jin K., Zeng H., Liu H., Chang Y. (2025). Natural killer cell infiltration elicits gender-dependent dichotomous clinical outcomes in urothelial carcinoma. Cancer.

[31] Aragaki A.K., Jing Y., Hoffman-Censits J., Choi W., Hahn N.M., Trock B.J., McConkey D.J., Johnson B.A. (2022). Gender-specific Stratification of Survival Following Immune Checkpoint Inhibitor Therapy Based on Intratumoral Expression of a B Cell Gene Signature. Eur. Urol. Oncol..

[32] Holm J.S., Funt S.A., Borch A., Munk K.K., Bjerregaard A.M., Reading J.L., Maher C., Regazzi A., Wong P., Al-Ahmadie H. (2022). Neoantigen-specific CD8 T cell responses in the peripheral blood following PD-L1 blockade might predict therapy outcome in metastatic urothelial carcinoma. Nat. Commun..

[33] Rijnders M., Nakauma-González J.A., Robbrecht D.G.J., Gil-Jimenez A., Balcioglu H.E., Oostvogels A.A.M., Aarts M.J.B., Boormans J.L., Hamberg P., van der Heijden M.S. (2024). Gene-expression-based T-Cell-to-Stroma Enrichment (TSE) score predicts response to immune checkpoint inhibitors in urothelial cancer. Nat. Commun..

[34] Klümper N., Sikic D., Saal J., Büttner T., Goldschmidt F., Jarczyk J., Becker P., Zeuschner P., Weinke M., Kalogirou C. (2022). C-reactive protein flare predicts response to anti-PD-(L)1 immune checkpoint blockade in metastatic urothelial carcinoma. Eur. J. Cancer.

[35] Sarfaty M., Golkaram M., Funt S.A., Al-Ahmadie H., Kaplan S., Song F., Regazzi A., Makarov V., Kuo F., Ostrovnaya I. (2023). Novel Genetic Subtypes of Urothelial Carcinoma with Differential Outcomes on Immune Checkpoint Blockade. J. Clin. Oncol..

[36] Song Y., Peng Y., Qin C., Wang Y., Yang W., Du Y., Xu T. (2023). Fibroblast growth factor receptor 3 mutation attenuates response to immune checkpoint blockade in metastatic urothelial carcinoma by driving immunosuppressive microenvironment. J. Immunother. Cancer.

[37] Banchereau R., Leng N., Zill O., Sokol E., Liu G., Pavlick D., Maund S., Liu L.-F., Kadel E., Baldwin N. (2021). Molecular determinants of response to PD-L1 blockade across tumor types. Nat. Commun..

[38] Kamoun A., de Reyniès A., Allory Y., Sjödahl G., Robertson A.G., Seiler R., Hoadley K.A., Groeneveld C.S., Al-Ahmadie H., Choi W. (2020). A Consensus Molecular Classification of Muscle-invasive Bladder Cancer. Eur. Urol..

[39] Sjödahl G., Lövgren K., Lauss M., Patschan O., Gudjonsson S., Chebil G., Aine M., Eriksson P., Månsson W., Lindgren D. (2013). Toward a Molecular Pathologic Classification of Urothelial Carcinoma. Am. J. Pathol..

[40] Rose T.L., Weir W.H., Mayhew G.M., Shibata Y., Eulitt P., Uronis J.M., Zhou M., Nielsen M., Smith A.B., Woods M. (2021). Fibroblast growth factor receptor 3 alterations and response to immune checkpoint inhibition in metastatic urothelial cancer: A real-world experience. Br. J. Cancer.

[41] Song Y., Jiang S., Peng Y., Qin C., Du Y., Xu T. (2024). Effect of FGFR alteration on prognosis in 1963 urothelial carcinoma patients with immune checkpoint inhibitors. Pharmacol. Res..

[42] Gupta S., Lee J.K., Mitchell J., Huang R.S.P. (2025). Fibroblast Growth Factor Receptor 3 Alteration Status and Outcomes on Immune Checkpoint Inhibitors in Patients with Metastatic Urothelial Carcinoma. JCO Precis. Oncol..

[43] Compérat E., Amin M.B., Epstein J.I., Hansel D.E., Paner G., Al-Ahmadie H., True L., Bayder D., Bivalacqua T., Brimo F. (2021). The Genitourinary Pathology Society Update on Classification of Variant Histologies, T1 Substaging, Molecular Taxonomy, and Immunotherapy and PD-L1 Testing Implications of Urothelial Cancers. Adv. Anat. Pathol..

[44] Boll L.M., Perera-Bel J., Rodriguez-Vida A., Arpí O., Rovira A., Juanpere N., de Oca S.V.M., Hernández-Llodrà S., Lloreta J., Albà M.M. (2023). The impact of mutational clonality in predicting the response to immune checkpoint inhibitors in advanced urothelial cancer. Sci. Rep..

[45] Afferi L., Cimadamore A., Gallioli A., Pradere B., Mertens L.S., Marcq G., Anguera G., Necchi A., Briganti A., Montorsi F. (2025). Tissue-based Biomarkers Steering Clinical Decisions in Patients with Urothelial Cancer. Eur. Urol..

[46] Mittal K., Joshi M. (2024). Perioperative immunotherapy in urothelial carcinoma: AMBASSADOR charts the path forward. Med.

[47] Cathomas R., Rothschild S.I., Hayoz S., Bubendorf L., Özdemir B.C., Kiss B., Erdmann A., Aeppli S., Mach N., Strebel R.T. (2023). Perioperative Chemoimmunotherapy with Durvalumab for Muscle-Invasive Urothelial Carcinoma: Primary Analysis of the Single-Arm Phase II Trial SAKK 06/17. J. Clin. Oncol..

[48] Conci N., Tassinari E., Tateo V., Rosellini M., Marchetti A., Ricci C., Chessa F., Santoni M., Grande E., Mollica V. (2024). How Do Molecular Classifications Affect the Neoadjuvant Treatment of Muscle-Invasive Urothelial Carcinoma?. Mol. Diagn. Ther..

[49] Makrakis D., Talukder R., Lin G.I., Diamantopoulos L.N., Dawsey S., Gupta S., Carril-Ajuria L., Castellano D., de Kouchkovsky I., Koshkin V.S. (2022). Association Between Sites of Metastasis and Outcomes with Immune Checkpoint Inhibitors in Advanced Urothelial Carcinoma. J. Clin. Genitourin. Cancer.

[50] Erlmeier F., Klümper N., Landgraf L., Strissel P.L., Strick R., Sikic D., Taubert H., Wach S., Geppert C.I., Bahlinger V. (2023). Spatial Immunephenotypes of Distant Metastases but not Matched Primary Urothelial Carcinomas Predict Response to Immune Checkpoint Inhibition. Eur. Urol..

[51] Esagian S.M., Khaki A.R., Diamantopoulos L.N., Carril-Ajuria L., Castellano D., De Kouchkovsky I., Park J.J., Alva A., Bilen M.A., Stewart T.F. (2021). Immune checkpoint inhibitors in advanced upper and lower tract urothelial carcinoma: A comparison of outcomes. BJU Int..

[52] Califano G., Ouzaid I., Laine-Caroff P., Peyrottes A., Collà Ruvolo C., Pradère B., Elalouf V., Misrai V., Hermieu J.F., Shariat S.F. (2022). Current Advances in Immune Checkpoint Inhibition and Clinical Genomics in Upper Tract Urothelial Carcinoma. Curr. Oncol..

[53] Su X., Lu X., Bazai S.K., Compérat E., Mouawad R., Yao H., Rouprêt M., Spano J.P., Khayat D., Davidson I. (2021). Comprehensive integrative profiling of upper tract urothelial carcinomas. Genome Biol..

[54] Chen Y., Fu J., Li Z., Chen Q., Zhang J., Yang Y., Yang P., Wang J., Liu Z., Cao Y. (2023). Cutoff values of PD-L1 expression in urinary cytology samples for predicting response to immune checkpoint inhibitor therapy in upper urinary tract urothelial carcinoma. Cancer Cytopathol..

[55] Bakaloudi D.R., Talukder R., Enright T., Leary J.B., Makrakis D., Diamantopoulos L.N., Hobeika C., Thomas V.M., Swami U., Zakopoulou R. (2025). Response and Survival with Immune Checkpoint Inhibitor in Patients with Advanced Urothelial Carcinoma and Histology Subtypes. Clin. Genitourin. Cancer.

[56] Jindal T., Zhang L., Deshmukh P., Reyes K., Chan E., Kumar V., Zhu X., Maldonado E., Feng S., Johnson M. (2023). Impact of Squamous Histology on Clinical Outcomes and Molecular Profiling in Metastatic Urothelial Carcinoma Patients. Clin. Genitourin. Cancer.

[57] Cerrato C., Crocerossa F., Marchioni M., Giannarini G., Gupta S., Albiges L., Brouwer O., Albersen M., Fankhauser C., Grimm M.O. (2024). Effect of Sex on the Oncological Outcomes in Response to Immunotherapy and Antibody-drug Conjugates in Patients with Urothelial and Kidney Cancer. Eur. Urol. Oncol..

[58] Incorvaia L., Scagliarini S., Marques Monteiro F.S., Takeshita H., Tapia J.C., Gandur Quiroga M.N., Lam E., Tural D., Popovic L., Campos-Gomez S. (2025). Sex as modifier of survival in patients with advanced urothelial cancer treated with pembrolizumab. Sci. Rep..

[59] Kawada T., Yanagisawa T., Mostafaei H., Sari Motlagh R., Quhal F., Rajwa P., Laukhtina E., von Deimling M., Bianchi A., Majdoub M. (2023). Impact of Performance Status on Oncologic Outcomes in Patients with Advanced Urothelial Carcinoma Treated with Immune Checkpoint Inhibitor. Eur. Urol. Focus.

[60] Rizzo A., Mollica V., Massari F. (2022). Expression of Programmed Cell Death Ligand 1 as a Predictive Biomarker in Metastatic Urothelial Carcinoma Patients. Eur. Urol. Focus.

[61] Coca Membribes S., Szabados B., Powles T. (2026). Towards biomarker-driven therapies for urothelial carcinoma. Nat. Rev. Clin. Oncol..

[62] Klümper N., Wüst L., Saal J., Ralser D.J., Zarbl R., Jarczyk J., Breyer J., Sikic D., Wullich B., Bolenz C. (2023). PD-L1 (CD274) promoter hypomethylation predicts immunotherapy response in metastatic urothelial carcinoma. Oncoimmunology.

[63] Loriot Y., Matsubara N., Park S.H., Huddart R.A., Burgess E.F., Houédé N., Banek S., Guadalupi V., Ku J.H., Valderrama B.P. (2023). Erdafitinib or Chemotherapy in Advanced or Metastatic Urothelial Carcinoma. N. Engl. J. Med..

[64] Bakaloudi D.R., Talukder R., Makrakis D., Diamantopoulos L., Enright T., Leary J.B., Patgunarajah U., Thomas V.M., Swami U., Agarwal N. (2024). Association of Tumor Mutational Burden and Microsatellite Instability With Response and Outcomes in Patients With Urothelial Carcinoma Treated With Immune Checkpoint Inhibitor. Clin. Genitourin. Cancer.

[65] Graf R.P., Fisher V., Huang R.S.P., Hamdani O., Gjoerup O.V., Stanke J., Creeden J., Levy M.A., Oxnard G.R., Gupta S. (2022). Tumor Mutational Burden as a Predictor of First-Line Immune Checkpoint Inhibitor Versus Carboplatin Benefit in Cisplatin-Unfit Patients with Urothelial Carcinoma. JCO Precis. Oncol..

[66] Ma Y.T., Hua F., Zhong X.M., Xue Y.J., Li J., Nie Y.C., Zhang X.D., Ma J.W., Lin C.H., Zhang H.Z. (2023). Clinicopathological characteristics and biomarker landscape for predicting the efficacy of PD-1/PD-L1 inhibitors in mismatch repair deficient urothelial carcinoma. Front. Immunol..

[67] Hammouda K., Tokuyama N., Corredor G., Pathak T., Dakarapu R., Genega E., Mian O.Y., Pavicic P.G., Diaz-Montero C.M., Mirtti T. (2025). AI-informed computational pathology classifier predicts outcomes across treatment modalities in muscle-invasive urothelial carcinoma. Cancer Lett..

[68] Nielsen T.J., Varga M.G., Cronister C.T., Ring B.Z., Seitz R.S., Ross D.T., Schweitzer B.L., McGregor K. (2023). The 27-gene IO score is associated with efficacy of PD-1/L1 inhibitors independent of FGFR expression in a real-world metastatic urothelial carcinoma cohort. Cancer Immunol. Immunother..

[69] Rizzo A., Zayed A., Vitale E., Buti S., Takeshita H., Crabb S., Roviello G., Seront E., Tapia J.C., Scagliarini S. (2025). Prognostic immunotherapy score (PIS) in patients with advanced urothelial carcinoma treated with pembrolizumab: Real-world data and validation from ARON-2 dataset. Clin. Exp. Metastasis.

[70] Penunuri A., Greenberg E., Phillips J., Katims A.B. (2026). The Emerging Role of B Cells and Tertiary Lymphoid Structures in Bladder Cancer. Curr. Urol. Rep..

[71] McGregor B.A., Sonpavde G. (2019). Enfortumab Vedotin, a fully human monoclonal antibody against Nectin 4 conjugated to monomethyl auristatin E for metastatic urothelial carcinoma. Expert Opin. Investig. Drugs.

[72] Sano T., Saito R., Aizawa R., Watanabe T., Murakami K., Kita Y., Masui K., Goto T., Mizowaki T., Kobayashi T. (2023). Current trends in the promising immune checkpoint inhibition and radiotherapy combination for locally advanced and metastatic urothelial carcinoma. Int. J. Clin. Oncol..

[73] Qiao Y., Zhao W., Gou Y., Li Y., Su F., Wang R., Wang J., Zhang H., Sun L., Qian F. (2025). Research hotspots and frontier analysis of the novel immune checkpoint Nectin-4. Hum. Vaccines Immunother..

[74] Nikolos F., Hayashi K., Hoi X.P., Alonzo M.E., Mo Q., Kasabyan A., Furuya H., Trepel J., Di Vizio D., Guarnerio J. (2022). Cell death-induced immunogenicity enhances chemoimmunotherapeutic response by converting immune-excluded into T-cell inflamed bladder tumors. Nat. Commun..

[75] Inoue S., Miszczyk M., Suleja A., Matsukawa A., Miyajima K., Dematteis A., Cormio A., Roessler N., Alfarhan A.R., Tsuboi I. (2025). Urinary biomarkers for immunotherapy response in urothelial carcinoma: Current status and future outlook. Expert. Rev. Mol. Diagn..

[76] Ma Q., Hou S., Ma H., Gao J., Song D. (2025). Prognostic significance of circulating tumor DNA in urothelial carcinoma patients undergoing immune checkpoint inhibitor therapy: A systematic review and meta-analysis. Front. Immunol..

[77] Duru G., van Egmond M., Heemskerk N. (2020). A Window of Opportunity: Targeting Cancer Endothelium to Enhance Immunotherapy. Front. Immunol..

[78] Febriyanto T., Muhammad F., Wijaya W., Oey O., Simadibrata D.M. (2024). Antibiotic use reduces the efficacy of immune checkpoint inhibitors in patients with urothelial carcinoma: A systematic review and meta-analysis. Urol. Oncol..

[79] Jang H.J., Hostetter G., Macfarlane A.W., Madaj Z., Ross E.A., Hinoue T., Kulchycki J.R., Burgos R.S., Tafseer M., Alpaugh R.K. (2023). A Phase II Trial of Guadecitabine plus Atezolizumab in Metastatic Urothelial Carcinoma Progressing after Initial Immune Checkpoint Inhibitor Therapy. Clin. Cancer Res..

